# Proteomics of Durum Wheat Grain during Transition to Conservation Agriculture

**DOI:** 10.1371/journal.pone.0156007

**Published:** 2016-06-09

**Authors:** Giovanna Visioli, Angelica Galieni, Fabio Stagnari, Urbana Bonas, Stefano Speca, Andrea Faccini, Michele Pisante, Nelson Marmiroli

**Affiliations:** 1 Department of Life Sciences, University of Parma, Parma, Italy; 2 Faculty of Bioscience and Technologies for Food, Agriculture and Environment, University of Teramo, Teramo, Italy; 3 Interdepartmental Measure Centre “Giuseppe Casnati,” University of Parma, Parma, Italy; 4 Regione Emilia-Romagna SITEIA, PARMA Technopole, Parma, Italy; Huazhong University of Science & Technology(HUST), CHINA

## Abstract

Nitrogen management in combination with sustainable agronomic techniques can have a great impact on the wheat grain proteome influencing its technological quality. In this study, proteomic analyses were used to document changes in the proportion of prolamins in mature grains of the newly released Italian durum wheat cv Achille. Such an approach was applied to wheat fertilized with urea (UREA) and calcium nitrate (NITRATE), during the transition to no-till Conservation Agriculture (CA) practice in a Mediterranean environment. Results obtained in a two-years field experiment study suggest low molecular weight glutenins (LMW-GS) as the fraction particularly inducible regardless of the N-form. Quantitative analyses of LMW-GS by 2D-GE followed by protein identification by LC-ESI-MS/MS showed that the stable increase was principally due to C-type LMW-GS. The highest accumulation resulted from a physiologically healthier state of plants treated with UREA and NITRATE. Proteomic analysis on the total protein fraction during the active phase of grain filling was also performed. For both N treatments, but at different extent, an up-regulation of different classes of proteins was observed: i) enzymes involved in glycolysis and citric acid cycles which contribute to an enhanced source of energy and carbohydrates, ii) stress proteins like heat shock proteins (HSPs) and antioxidant enzymes, such as peroxidases and superoxide dismutase which protect the grain from abiotic stress during starch and storage protein synthesis. In conclusion N inputs, which combined rate with N form gave high yield and improved quality traits in the selected durum wheat cultivar. The specific up-regulation of some HSPs, antioxidant enzymes and defense proteins in the early stages of grain development and physiological indicators related to fitness traits, could be useful bio-indicators, for wheat genotype screening under more sustainable agronomic conditions, like transition phase to no-till CA in Mediterranean environments.

## Introduction

Durum wheat (*Triticum turgidum* L. subsp. *durum* (Desf.) Husn.) is one of the most important crops in the world and a major food source, unique for pasta production, the Mediterranean countries representing around 75% of the world durum wheat growing area. Wheat contributes a significant source of carbohydrates, though its protein and micronutrient contents have raised a renewed interest in whole meal durum-based products. Wheat kernel proteins are classified according to their solubility properties into prolamins as gliadins, high molecular weight glutenins (HMW-GS) and low molecular weight glutenins (LMW-GS) soluble in diluted acid or alkali or alcohol-water mixtures, and total proteins including albumins, globulins and metabolic enzymes which are water and salt soluble [1]. Gluten proteins represent about 80% of wheat seed proteins, and are the most important determinant of the dough properties. Other proteins are less abundant in mature grain but are rich in essential aminoacids lysine, tryptophan and methionine, which are very important for human health, whereas metabolic enzymes have important roles in protein folding and polymerization during grain filling influencing also quality traits [2]. The amount of durum wheat proteins in grains is strictly associated with the environmental conditions, especially during the grain filling period [3]. In this context, soil nitrogen (N) availability plays a major role [4]; high N fertilization conditions have been demonstrated to increase synthesis and accumulation of gluten proteins [5,6]. N fertilization also modulates the accumulation of total proteins as metabolic enzymes and consequently the synthesis of starch and storage protein during grain filling [7]. Thus, modulation of the accumulation of prolamins as far as proteins and enzymes involved in storage protein synthesis and carbohydrate accumulation during grain filling should be considered in N fertilization strategies to obtain high quality traits.

Soil management is another fundamental variable influencing quality traits, in particular protein content and profile, as well as yield, especially in Mediterranean regions characterized by low and erratic rainfall and by soils with low organic matter and N contents, where durum wheat is a major crop. Adoption of Conservation Agriculture (CA), which imply no or minimum soil disturbance, soil mulch cover and diversified cropping system, may allow a more sustainable agricultural production, mitigating the negative effects of soil fertility losses and climate changes [8]. However, the transition to no-till CA represents an holistic change in management which requires an adaptation at the individual farm-level [8]. During this phase which can last about 10 years, the short-time decline of yields is observed, due to higher annual crops weed, pest, and disease pressures which may increase over time with continuous no-till systems [9]. The soil starts rebuilding aggregates although measurable changes in the soil carbon content are not expected; moreover, crop residues production is sometimes low, especially in dry environments [10]. In such unstable conditions, N fertilization strategies of cereal crops require adjusting rates, timing, splitting, and source of applications, considering also that only very few studies are available with results often inconsistent [11]. However, the individual effects of residue retention and crop rotation reduce the negative impacts of no-till, especially in dry climates with positive effects on cereal growth, soil N, physical properties, moisture and organic matter [12,13]; such conditions would lead to a reduction in the required N inputs.

The grain protein content of durum wheat at harvest is related to plant N status at anthesis [14]. Thus, vegetation indices could be very useful to monitor plant fitness and plant N content during the vegetative phase and, consequently, to predict quality traits at harvest [14,15].

In a previous study [16], it was demonstrated that 150 Kg N ha^-1^ is the rate that allows both yield and gluten proteins accumulation to be maximized for the newly-released Italian cultivar Achille, during transition to no-till CA. In this paper, starting from such a N rate we want to: i) test the effects of N-form (urea and calcium nitrate) on single prolamin components in mature grain by 2D-GE and ESI-LC-MS/MS proteomic analyses, to evidence possible single protein modulations in response to N fertilization treatment; ii) analyse by the same comparative proteomic approach the effect of N-form on total proteins in developing grain during the active phase of grain filling and iii) correlate the data with physiological indicators measured during vegetative growth. The results obtained suggest that N fertilization treatments, giving the highest wheat yield during the transition to no-till CA, increase quality traits as gluten proteins at harvesting and are of fundamental importance for targeting wheat quality by conventional breeding or genetic engineering to specific end-users.

## Material and Methods

### Field trial description, plant sampling and physiological traits

*Triticum turgidum* L. subsp. *durum* (Desf.) Husn., cv ‘Achille’ was utilized in this study. This cultivar was chosen for experimental purpose because of its particular adaptability to Mediterranean environments. The field trials were carried out during the seasons 2010–2011 and 2011–2012 (referred to below as 2011 and 2012, respectively) at the experimental field of the University of Teramo (Mosciano Sant’Angelo, Italy, 42° 42’ N, 13° 52’ E, 101 m a.s.l.), during the first two years of transition to no-till CA. The previous crops were coriander (*Coriandrum sativum* L.) in 2011 and durum wheat in 2012, which were harvested at the end of July and at the middle of June, respectively. Crop residues were kept on the soil surface and homogenously distributed to obtain an uniform layer of mulch. Durum wheat was sown on mid-November (8/11/2010 and 17/11/2011) by direct seeding (Gaspardo Direttissima, Gruppo Maschio Gaspardo SpA, Campodarsego, PD, Italy), at a rate of 350 viable seeds m^-2^. Descriptions of the field agricultural management practices are provided in more details in our previous work [16]. Average minimum and maximum temperatures and total rainfall for the trials are reported in S1 Table. Starting from our previous results on comparative analysis of two N fertilizer forms (urea and calcium nitrate) applied at four N application rates (50, 100, 150 and 200 kg N ha^-1^) [16], here it was analysed only the data related to urea and calcium nitrate at 150 kg N ha^-1^ (named UREA and NITRATE, respectively), the rate which maximized yield and grain protein content. Such N rate is supported by other studies performed in Southern Italy, under a no-till system [17]. Unfertilized plots were added as CONTROL. UREA was selected as it is the most widely used form of N fertilizer applied to agroecosystems [18], characterised by slow N release; NITRATE (NO_3_-based N fertilizer) was selected because of its lower N_2_O emissions as compared to NH_4_-based N fertilizers [19, 20], characterised by prompt N release. According to the usual practices in the specific area and soil conditions, the N fertilizers were split into pre-sowing (20% of the total amount) and cover dressings (tillering, 40%, and 4^th^ node detectable, 40%; DC22 and DC34, respectively). The phenological stages were monitored on 20 randomly tagged plants per plot and were scored following the Zadoks Decimal Code [21]. A growth stage (DC) was assigned when it was reached by 50% of the monitored plants.

Starting from the ‘medium milk’ phenological stage (DC75), 10 whole main wheat shoots within each experimental unit were randomly collected, as reported in S2 Table; in total, 4 sampling dates (corresponding to DC75, DC77, DC85 and DC87) were considered. Plants were separated into leaves, stems and spikes. Kernels were collected by hand threshing and grain (mg DW spike^-1^), leaves and stems dry weights (mg DW plant^-1^) was determined after oven drying at 80°C, until constant weight. Grains from 10 additional spikes were stored at -20°C for protein analysis. At harvest plant height, ear length, ripe yield, grain N content and total N in the above-ground biomass (leaves and stems) were measured; the N content in kernels was converted to grain protein concentration (GPC, %) through the factor of 5.75 [16]. Grain nitrogen utilization efficiency (grain-NutE; kg-DM kg-N^-1^) was calculated as the grain dry matter (DM) yield divided by all the N in the above-ground parts of the crop at maturity [22].

Chlorophyll content was estimated by SPAD (soil-plant analysis development) with a 502 plus portable chlorophyll meter (Konica Minolta, Inc., Tokyo, Japan). For each experimental unit, measurements were taken on the mid-section of 10 fully-expanded and sun-oriented flag leaves at the phenological stages of DC65, DC71, DC75, DC77 and DC83 (corresponding to 0, 8, 15, 23 and 26 DPA in 2011 and to 0, 6, 13, 21 and 24 DPA in 2012).

In 2012, a HandHeld 2 Pro Portable FieldSpec Spectroradiometer (ADS Inc., Boulder, CO, USA) was used to measure the reflected light from the canopy. Readings were taken under clear sky conditions, starting from anthesis and for a total of 4 sampling dates (corresponding to DC65, DC71, DC75 and DC77–0, 6, 13 and 21 DPA).

Starting from the canopy’s reflectance data, the normalized difference vegetation index (NDVI), green normalized difference vegetation index (GNDVI), optimized soil-adjusted vegetation index (OSAVI), simple ratio (SR), structure insensitive pigment index (SIPI), nitrogen reflectance index (NRI), modified chlorophyll absorption ratio index (MCARI), triangular vegetation index (TVI) and water index (WI) were calculated as follows:

NDVI=(ρNIR–ρRED)/(ρNIR+ρRED) [23]

GNDVI=(ρ750-ρ550)/(ρ750+ρ550) [24]

OSAVI=(1+0.16)(ρ800–ρ670)/(ρ800+ρ670+0.16) [25]

SR=(ρ800-ρ900)/(ρ650–ρ700) [26]

SIPI=(ρ800-ρ445)/(ρ800-ρ680) [27]

NRI=(ρ570-ρ670)/(ρ570+ρ670) [28]

MCARI=[(ρ700-ρ670)-0.2)x(ρ700–ρ550)]x(ρ700/ρ670) [29]

TVI=0.5x[120x(ρ750-ρ550)-200x(ρ670-ρ550)] [30]

$WI=\rho_{}900_{}\;/\;\rho_{}970$ [31]

### Prolamin and total protein extractions

Thirty-five g of mature grains (DC92) from each treatment in 2011 and 2012 were crushed with Knifetec TM 1095 (Foss, Hillerød, Denmark) to obtain a fine powder to analyze the composition of gliadin, HMW-GS and LMW-GS fractions. Gluten proteins were extracted from wheat flour (30 mg) with the sequential procedure of Singh and collaborators [32]. In brief, fine powder was extracted with 1.5 mL of 55% (v/v) propan-2-ol for 20 min with continuous mixing at 65°C, followed by centrifugation for 5 min at 10,000 rpm. This step was repeated three times in total and the gliadin components were extracted. HMW and LMW-GS fractions were extracted from the pellet and all fractions were quantified as previously described [33]. Total gluten protein content (GLUTEN) was calculated by summing the contents of all the single gluten fractions (gliadins, HMW and LMW-GS). Three biological replicates were prepared for each sample.

For total protein extraction, at the DC75 phenological stage (15 days post-anthesis (DPA) and 13 DPA in 2011 and 2012, respectively), 150 mg of immature seeds were ground to a fine powder in liquid nitrogen and the powder was then suspended in cold acetone containing 10% (w/v) trichloroacetic acid (TCA) and 0.07% (v/v) β-mercaptoetanol, 0.4% proteinase inhibitor cocktail (Sigma Aldrich), vortexed and kept at -20°C overnight. Each sample was centrifuged at 20000 g for 15 min at 4°C and the resulting pellet was washed twice by re-suspending in cold acetone containing 0.07% (v/v) β-mercaptoetanol, 0.4% proteinase inhibitor cocktail (Sigma Aldrich) for 1 h each at -20°C before further centrifugation at 20000 g for 15 min at 4°C. The resulting pellet was vacuum dried for 30 min, and solubilized in freshly-prepared buffer containing 7 M Urea, 2 M thiourea, 4% (w/v) 3-[(3-cholamidopropyl)dimethyl-ammonio]-1-propanesulfonate (CHAPS), 18 mM TrisHCl pH 8, 0.4% proteinase inhibitor, 14 mM DTT and incubated 20 min on ice. Each sample was then centrifuged at 35000 g 25 min at 4°C and the supernatant was re-centrifuged at 35000 g 25 min at 4°C. The supernatant containing protein samples was quantified and aliquots of 500 μg precipitated in 4 volumes of cold acetone and left at -20°C over-night.

### Two-dimensional gel electrophoresis (2D-GE)

In 2011 and 2012, 2D-GE separation was carried out on both LMW-GS and total protein extracts from developing grains. In particular for the LMW-GS fraction, immobiline Dry-Strip (7 cm, GE Healthcare Europe GmbH) pH 6–11 was rehydrated with 85 μL of rehydration solution containing 9 M urea, 4% (w/v) CHAPS, 2% (v/v) immobilised pH gradient (IPG) buffer pH 6–11 (GE Healthcare), 1% (w/v) bromophenol blue and 1.2% (v/v) DeStreak Reagent (GE Healthcare). The rehydration step was carried out for 12 h at 20°C. After that, 25 μg of Savant dried LMW-GS were dissolved in 50 μL of a solution containing 9 M urea, 4% (w/v) CHAPS, 2% (v/v) IPG buffer pH 6–11 (GE Healthcare), 1% (w/v) bromophenol blue, 20 mM DTT and loaded with cup loading procedure at the anodic end of the strip. The iso-electric focusing (IEF) was carried out at 20,200 V/h in a Protean i12 IEF System (Bio-Rad, Hercules, CA).

Five hundred μg of total proteins were suspended into 185 μL of rehydration solution containing 7 M urea, 2M Thiourea, 4% (w/v) CHAPS, 0.4% (v/v) IPG buffer pH 4–7 (Biorad), 0.1% (w/v) bromophenol blue, 1.6% (v/v) DeStreak Reagent (GE Healthcare) and loaded into immobiline Dry-Strip (11 cm, Biorad) pH 4–7. After 12 h passive rehydration, IEF was carried out at 40,000 V/h in in a Protean i12 IEF System (Bio-Rad).

Both 7 cm and 11 cm strips were then equilibrated 15 min in equilibration buffer containing 6 M urea, 2% (w/v) SDS, 75 mM Tris-HCl pH 8.8, 29.3% (v/v) glycerol, 1% (w/v) bromophenol blue with 1% (w/v) DTT, and then in the same buffer containing 2.5% (w/v) iodoacetamide (IAA) for an additional 15 min. The second-dimension was performed on Mini-PROTEAN Tetra Cell (Bio-Rad) 12% acrylamide GTX precast gels (Bio-Rad) for 7 cm strips and on Criterion Dodeca Cell (Bio-Rad) 12% acrylamide Criterion XT precast gels for 11 cm strips. Molecular Weight Marker (Mw 14,000–66,000) (Sigma Aldrich) was utilised. Three biological replicates were obtained by sequential extractions of the same batch of seeds (see section Protein extraction and quantification).

### Spot digestion and LC-ESI-MS/MS

Spots were removed from the 2D-GE with a razor blade and destained in a solution of 100 mM 1:1 (v/v) ammonium bicarbonate/acetonitrile (ACN) overnight. In-gel digestion was performed with 12.5 ng/mL chymotrypsin. The digested peptides were then suspended in 10 μL of 0.1% TFA and purified with a ZipTipC18 (Merck Millipore, Billerica MA, USA) using the procedure recommended by the manufacturer.

After digestion, peptides were injected into the mass spectrometer: HPLC system DIONEX Ultimate 3000 coupled with a LTQ Orbitrap XL equipped with a pneumatically-assisted ESI interface (Thermo Fisher Scientific). The system was controlled by the Thermo Scientific Xcalibur software. Samples were suspended in 50 μL of 0.1% (v/v) formic acid in water (solvent A). Injection volume was set to 5 μL. Samples were carried into the μ-Precolumn Cartridge, Acclaim PepMap100 C18 (5 mm length x 0.3 mm internal diameter, 5 μm particle size, 100 A pore size) (Thermo Fisher Scientific) with an isocratic flow 30 μL/min of 2% ACN with 0.08% (v/v) formic acid (solvent B) for 30 sec.

Peptides were separated on a reverse phase HPLC chromatographic column, Jupiter C18 (150 mm×0.3 mm, 5 μm particle size, 300 A) (Phenomenex, Torrance, CA, USA) using an ACN gradient as follow: Solvent B for 4 min, 5–50% in 56 min and 50–95% in 2 min at flow rate of 4 μL/min. Solvent B was maintained at 95% for 10 min before column re-equilibration (15 min). Mass parameters: sheath gas rate (nitrogen, 99.99% purity), 8 arbitrary units; ESI voltage, 3.5 kV; capillary voltage, 30 V; capillary temperature, 275°C; tube lens, 110 V; Collision Induced Dissociation (CID), 35. Data dependent acquisition (DDA) mode: mass range, 250–2,000 amu; charge state rejection, *z* = 1; ion count threshold, 50,000.

### Statistical analysis

The dynamics of leaves, stems and grains dry weights and of estimated chlorophyll content over thermal time after anthesis were analysed with a split-plot ANOVA; treatments were considered as main factor and thermal time regarded as secondary factor. The contents of gluten protein fractions in grains at maturity as well as plant height, ear length and grain-NutE were subjected to one-way ANOVA. Means separation was performed through Fisher’s Least Significant Difference (LSD) test. Before ANOVA, data were analysed to test the adequacy of normality and homoscedasticity assumptions; such assumption were satisfied, therefore any data transformation was not applied. The correlations between GPC and GLUTEN at harvest with spectral vegetation indices (VI*s*: NDVI, GNDVI, OSAVI, SR, SIPI, NRI, MCARI, TVI and WI) were tested by Pearson’s correlation; data were previously subjected to Shapiro-Wilk normality test. Statistical analyses were performed using R software [34]. Principal component analysis (PCA) was applied to interpret and summarize the association between treatments and variables (vegetation indices) and the correlation among VI*s*. Twelve treatments (CONTROL_0DPA, CONTROL_6DPA, CONTROL_13DPA, CONTROL_21DPA, UREA_0DPA, UREA_6DPA, UREA_13DPA, UREA_21DPA, NITRATE_0DPA, NITRATE_6DPA, NITRATE_13DPA and NITRATE_21DPA), obtained as a combination of the 3 N treatments (CONTROL, UREA and NITRATE) at four sampling dates during grain development (0, 6, 13 and 21 DPA) in 2012, were tested. PCA was performed using the Excel add-in Multibase 2015 package (Numerical Dynamics, Japan).

Image analysis of the 2D-GE gels (three replicates was performed for each biological replicate and treatment considered) was carried out using PDQuest 8.0 2D Analysis Software (Bio-Rad). Each gel was analysed for spot detection, background subtraction, and protein spot OD intensity quantification. For LMW-GS quantification, the intensity (integrated optical density, IOD) of the same number of clearly visible spots was normalized to the total LMW-GS protein fraction. Data were subjected to analysis of variance (one-way ANOVA); comparisons of normalized mean spot volumes between treatments were performed using Fisher’s LSD test. For the total protein fraction, the gel image showing the highest number of spots and the best protein pattern was chosen as a reference template. Spots were analysed and compared by co-ordination with the reference template. Gels were divided into three groups (CONTROL, UREA and NITRATE treatments) and for each protein spot the average spot quantity value and its variance coefficient in each group were determined. Statistical analysis (Student’s t test) was performed to identify proteins that were significantly (p<0.05) increased or decreased in the three sets of samples.

## Results and Discussion

### Biomass dynamics, agronomic traits, total gluten proteins in mature grains and physiological parameters

**Fig 1:**
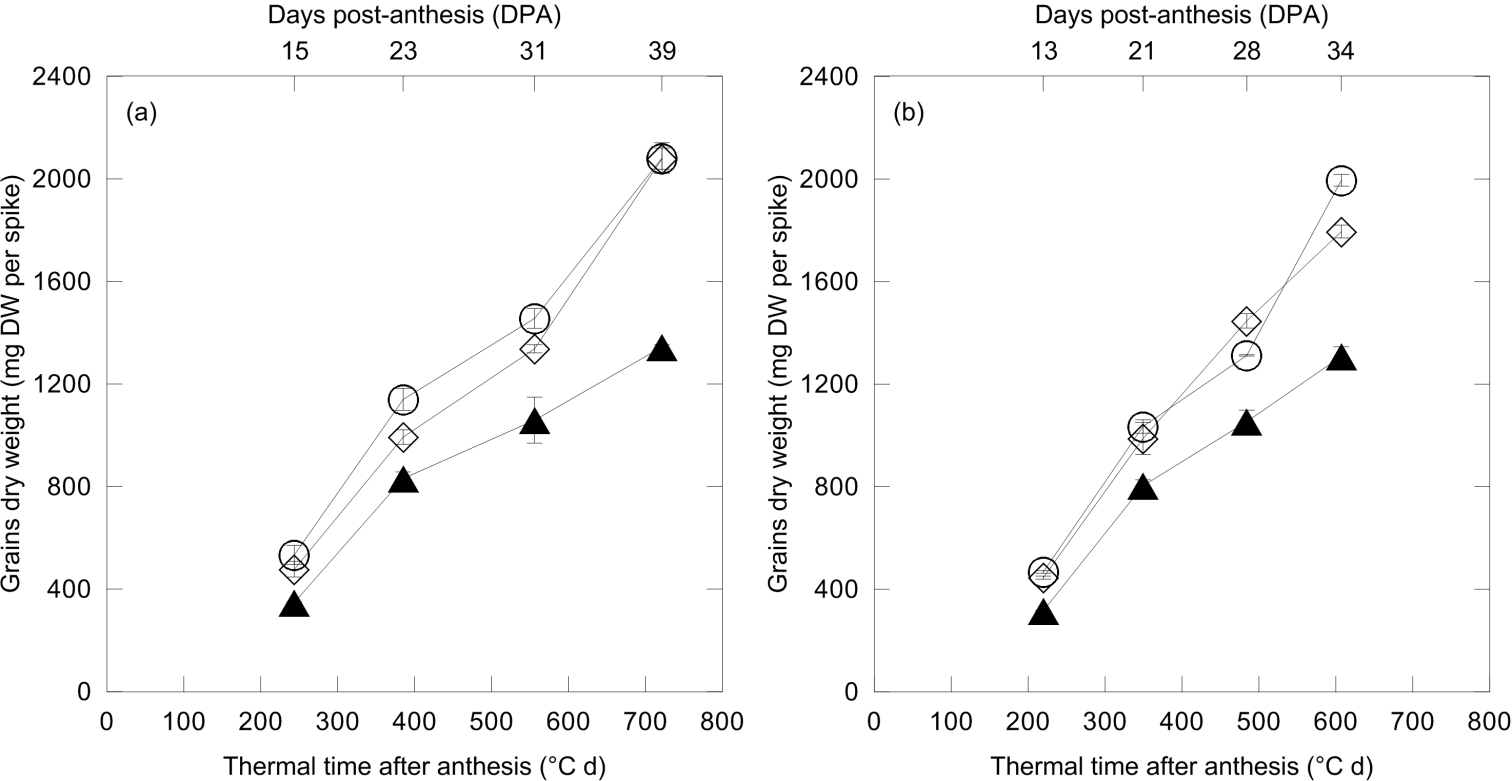
Changes of grain dry weight (mg DW per spike) against thermal time after anthesis (cumulative average daily air temperature exceeding 0°C, °C d) and days post-anthesis (DPA) (above axis) of durum wheat in 2011 (a) and 2012 (b). Wheat plants were exposed to control conditions (unfertilized CONTROL, black triangle) and to two N fertilization treatments with urea (UREA, white rhombus) and calcium nitrate (NITRATE, white circle) at the rate of 150 kg N ha^-1^ (mean value ± standard errors, n = 3 independent replicates; split-plot ANOVA over thermal time after anthesis; effects: (a) *treatment*, P < 0.01; *time*, P < 0.01; *treatment x time*, P< 0.01; (b) *treatment*, P < 0.01; *time*, P < 0.01; *treatment x time*, P< 0.01).

In both growing seasons, a similar trend for grain dry mass accumulation during grain-filling was observed for all treatments (Fig 1); the two fertilizer treatments enhanced significantly grain DW per spike with respect to the CONTROL by 29% and 24% in 2011 and 2012, respectively (average over both N-form and thermal time). Greater differences between UREA and NITRATE applications were recorded in 2012 only at the hard dough phenological stage (DC87), with NITRATE inducing a significantly higher grain DW (1795 vs. 1995 mg DW per spike).

**Fig 2:**
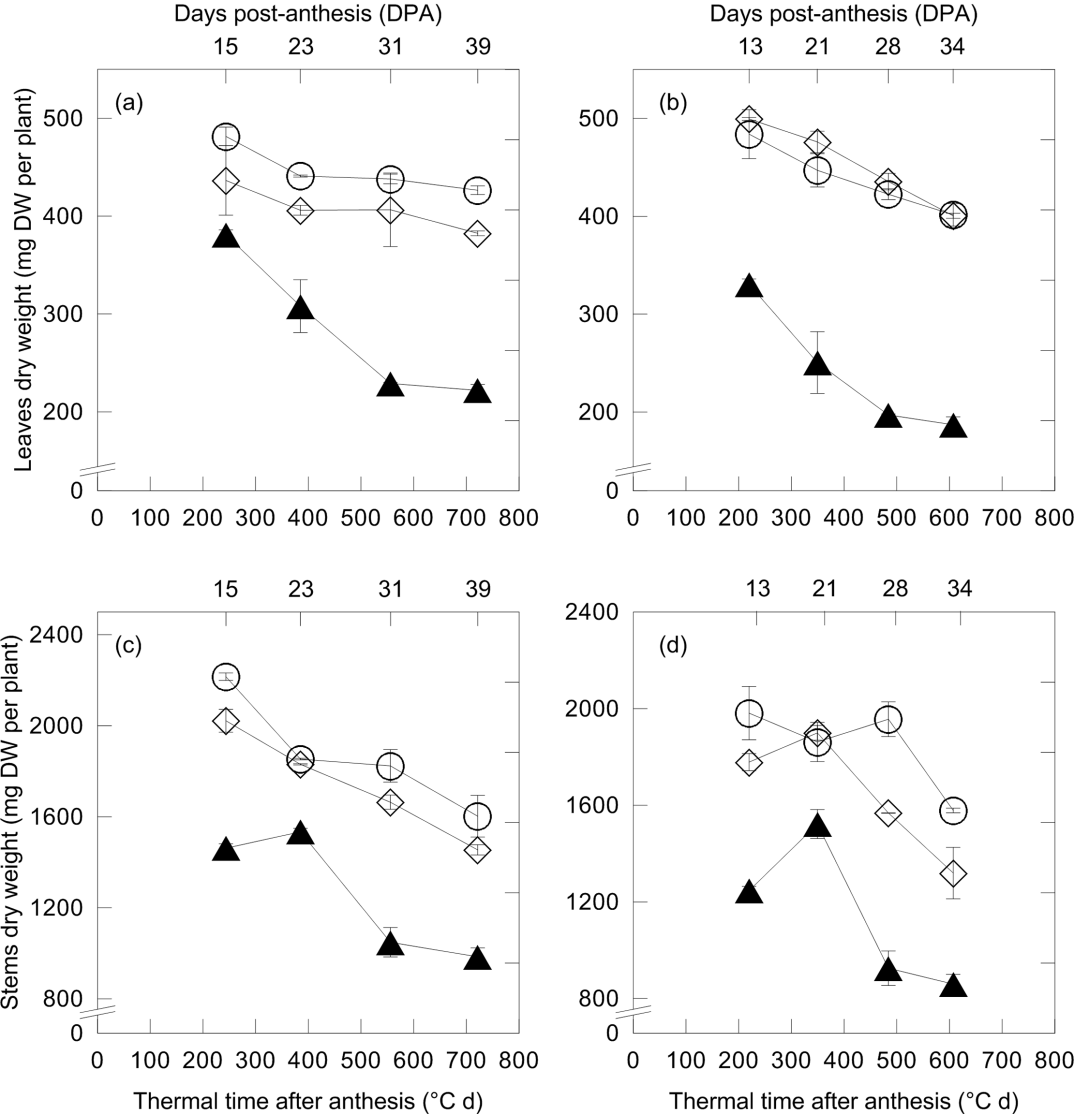
Changes of leaves dry weight (mg DW per plant) and stems dry weight (mg DW per plant) against thermal time after anthesis (cumulative average daily air temperature exceeding 0°C, °C d) and days post-anthesis (DPA) (above axis) of durum wheat in 2011 (a and c, respectively) and 2012 (b and d, respectively). Wheat plants were exposed to control conditions (unfertilized CONTROL, black triangle) and to two N fertilization treatments with urea (UREA, white rhombus) and calcium nitrate (NITRATE, white circle) at the rate of 150 kg N ha^-1^ (mean value ± standard errors, n = 3 independent replicates; split-plot ANOVA over thermal time after anthesis; effects: (a) *treatment*, P < 0.01; *time*, P < 0.01; *treatment x time*, P = 0.08; (b) *treatment*, P < 0.01; *time*, P < 0.01; *treatment x time*, P = 0.33; (c) *treatment*, P < 0.05; *time*, P < 0.01; *treatment x time*, P < 0.01; (d) *treatment*, P < 0.05; *time*, P < 0.01; *treatment x time*, P < 0.05).

The response of the crop in terms of above-ground biomass production to N fertilization was similar in 2011 and 2012 growing seasons (Fig 2). Both N-forms significantly increased leaves and stems dry weights with respect to unfertilized CONTROL; differences between N-treatments were not significant (Fig 2, LSD: (a) 0.607; (b) 0.510; (c) 1.302; (d) 1.981). Such trend observed during grain-filling period, was confirmed by plant height and ear length values at harvest (Table 1).

**Table 1:** Plant height (cm), ear lenght (cm) and grain nitrogen utilization efficiency (grain-NutE; kg- DM kg-N^-1^) as recorded at harvest, in 2011 and 2012. Wheat plants were exposed to control conditions (unfertilized CONTROL) and to two N fertilization treatments with urea (UREA) and calcium nitrate (NITRATE) at the rate of 150 kg N ha^-1^. Data are averages ± standard errors, for n = 3 independent replicates. Different letters indicate significant differences at p<0.05 (Fisher’s LSD test).

	*2011*			*2012*		
*Treatment*	Plant height (cm)	Ear length (cm)	Grain-NutE (kg-DM Kg-N^-1^)	Plant height (cm)	Ear length (cm)	Grain-NutE (kg-DM Kg-N^-1^)
CONTROL	64.1 ± 0.61 b	4.5 ± 0.18 b	63.4 ± 0.08 a	61.9 ± 0.55 b	4.0 ± 0.02 b	65.5 ± 2.05 a
UREA	75.4 ± 2.33 a	5.8 ± 0.01 a	34.6 ± 1.35 b	70.6 ± 0.51 a	6.6 ± 0.41 a	35.6 ± 0.47 b
NITRATE	75.0 ± 2.43 a	5.9 ± 0.16 a	38.1 ± 0.71 b	71.7 ± 0.22 a	6.7 ± 0.40 a	45.8 ± 1.99 b
*F test*	*	*	**	**	*	*

*P < 0.05

**P < 0.01; ns = not-significant.

**Fig 3:**
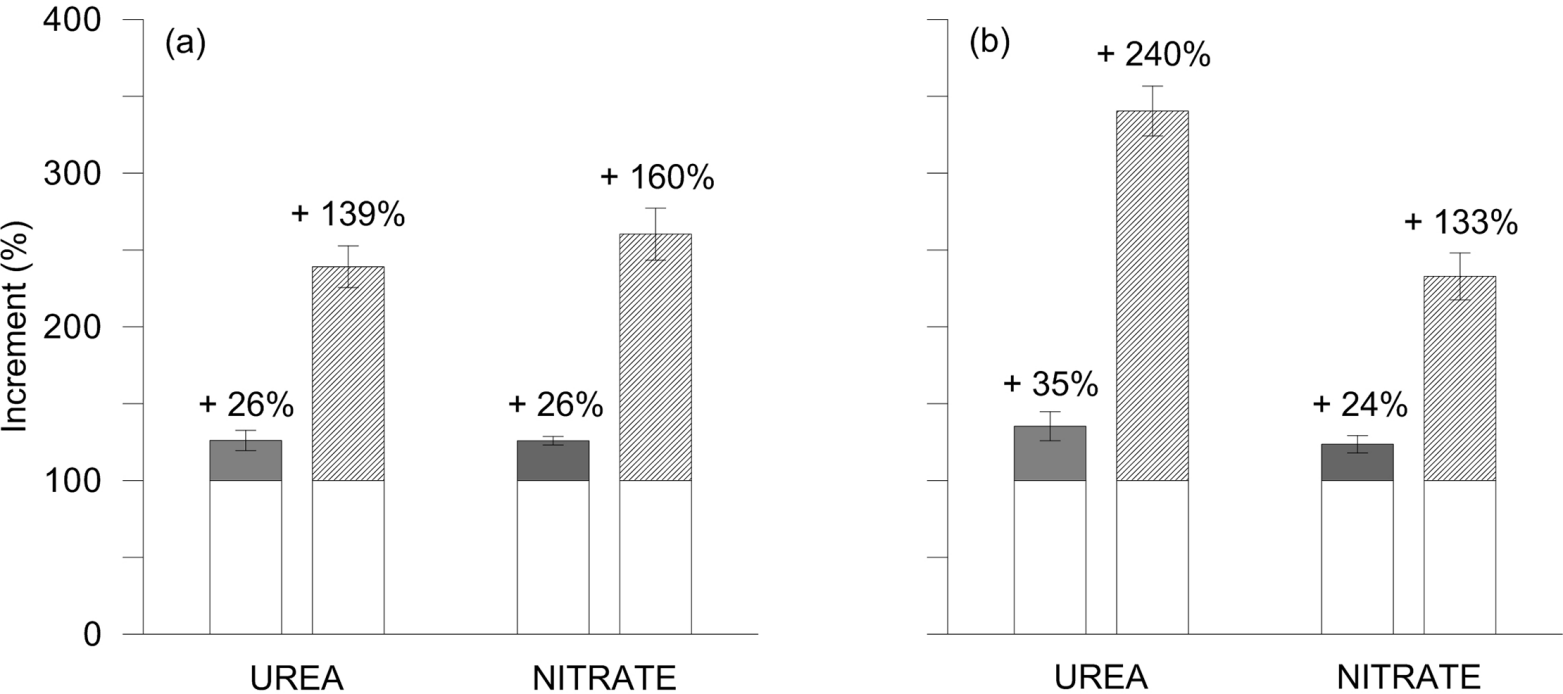
Increment of gliadins and glutenins in durum wheat mature grains under UREA and NITRATE fertilisation treatments respect to CONTROL. Percentage increments of gliadins (grey chart) and glutenins (diagonal lines; LMW-GS plus HMW-GS fractions) with respect to CONTROL (white chart) in durum wheat fertilized with urea (UREA) and calcium nitrate (NITRATE) at the rate of 150 kg N ha^-1^ in 2011 (a) and 2012 (b). Data are averages ± standard errors, for n = 3 independent replicates.

Gluten protein contents in mature grains increased due to N availability, reaching its maximum at 150 kg N ha^-1^ [16]. At this N-rate both N-forms affected the accumulation of glutenins with respect to gliadins (150% and 187% increase—averaged over N-form—in 2011 and 2012, respectively; Fig 3), according to previous data, suggesting differences in the expression of various gluten genes [35].

**Fig 4:**
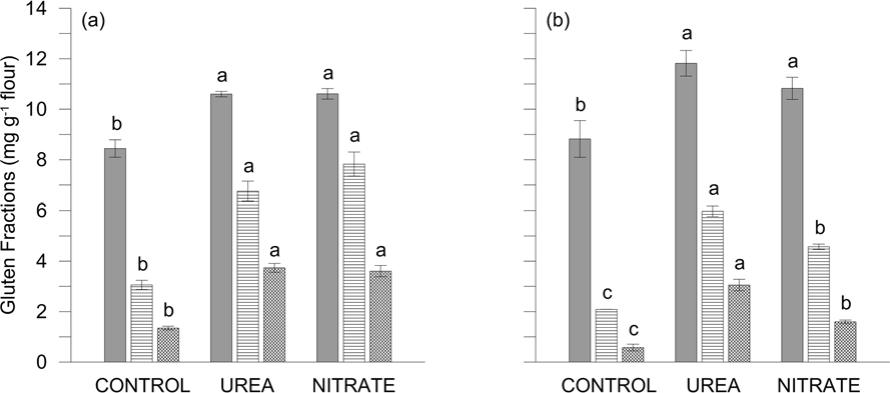
Gliadin and glutenin contents in in durum wheat mature grains under CONTROL and UREA and NITRATE fertilisation treatments. Gliadins (mg g^-1^ flour; grey chart) and glutenin (GS) fractions (mg g^-1^ flour; horizontallines: LMW-GS fraction; diagonal cross: HMW-GS fraction) in durum wheat grains fertilized with urea (UREA) and calcium nitrate (NITRATE) at the rate of 150 kg N ha^-1^ plus unfertilized CONTROL in 2011 (a) and 2012 (b). Data are averages ± standard errors, for n = 3 independent replicates. Different letters indicate significant differences at p<0.05 (Fisher’s LSD test).

The higher accumulation of glutenins in response to N-fertilization results in a lower gliadin:GS ratio, of interest, because the ratio of monomeric vs. polymeric gluten proteins constitutes a criterion for high quality traits of durum wheat. However, the effects of UREA and NITRATE on single gluten fractions (gliadin, HMW-GS and LMW-GS) showed year-to-year differences (Fig 4). Total glutenins were significantly lower, by about 30%, in 2012, while gliadin contents were relatively stable across years (Fig 4). This is probably attributable to a late water stress occurred in 2012 (70.8 vs. 47.6 mm of rainfall during grain filling in 2011 and 2012, respectively) which was confirmed by the estimation of crop water status, starting from 23 and 21 days post anthesis (DPA) in 2011 and 2012, respectively (DC77 phenological stage). Indeed in 2012, averaging over N treatments and phenological stages (DC77 and DC83—data not shown), canopy temperature (CT) and stomatal conductance (POR) gave higher and lower values (CT: 23.4 and 27.1°C; POR: 99.2 and 69.2 mmol m^-2^ s^-1^ in 2011 and 2012), respectively. Protein components are very sensitive to drought during the later phenophases of the filling period [36] and, as the maximum rate of synthesis of glutenins takes place later than that of gliadins [37], we observed a significant decline of glutenin accumulation [38]. Differences between the two N-forms occurred only in 2012 with UREA inducing significantly higher values for both GS fractions (Fig 4). This is explained by the higher temperatures registered during grain-filling period, which reduced grain carbohydrate accumulation rather than N accumulation, confirming previous studies [39] and also explaining the differences in terms of grain DW and yield [16], as previously observed [40]. To support these results, we have measured the grain-NutE, which represents the yield per unit of N-uptake (Table 1). Despite differences between the two N-forms were not significant, we observed higher grain-NutE values for NITRATE rather than UREA, especially in 2012 (35.6 vs 45.8 kg-DM kg-N^-1^ for UREA and NITRATE, respectively). Indeed, there is an inverse relationship between grain-NutE and grain %N—as demonstrated across different crop species [41]—and high-quality wheat can be expected to have a low grain-NutE [22]. Moreover, we also observed a clear inverse relationship between grain-NutE and total GS in grains, both in 2011 and 2012 (Pearson’s correlation coefficients: 2011, -0.95; 2012, -0.99).

**Fig 5:**
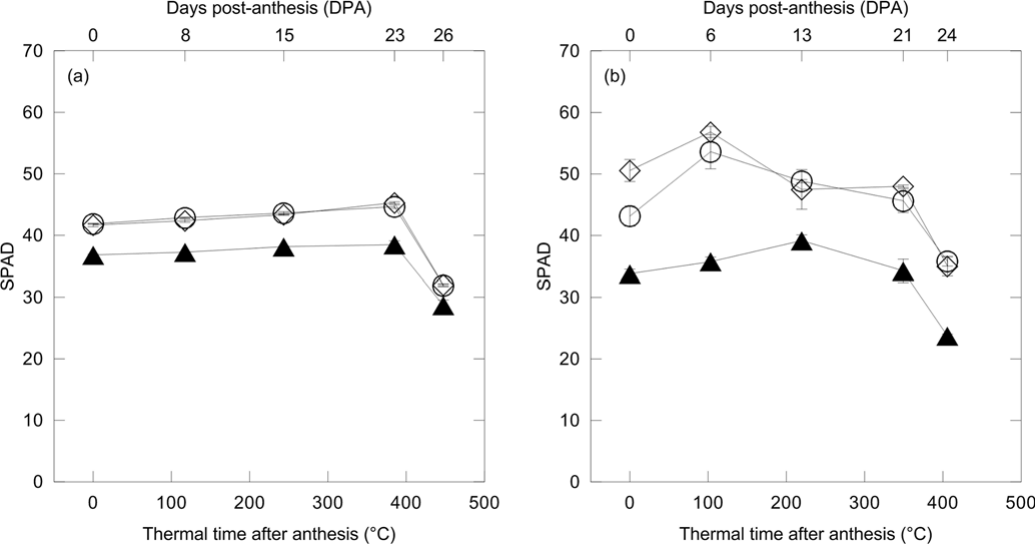
Changes of soil-plant analysis development (SPAD) against thermal time after anthesis (cumulative average daily air temperature exceeding 0°C, °C d) and days post-anthesis (DPA) (above axis) of durum wheat in 2011 (a) and 2012 (b). Wheat plants were exposed to control conditions (unfertilized CONTROL, black triangle) and to N fertilization treatments with urea (UREA, white rhombus) and calcium nitrate (NITRATE, white circle) at the rate of 150 kg N ha^-1^ (mean value ± standard errors, n = 3 independent replicates; split-plot ANOVA over thermal time after anthesis; effects: (a) *treatment*, P < 0.01; *time*, P < 0.01; *treatment x time*, P < 0.05; (b) *treatment*, P < 0.01; *time*, P < 0.01; *treatment x time*, P< 0.01).

To correlate the effect on N fertilization with the kinetics of protein accumulation, the physiological status of the crop during grain development was monitored. SPAD significantly discriminated the unfertilized CONTROL from both fertilized treatments (Fig 5). The greater chlorophyll (Chl) content in leaves of N-fertilized plants, as estimated by SPAD, is likely to have improved plant photosynthetic activity, favoring the accumulation of N and assimilates in developing kernels. Indeed, as previously stated by Matsunaka et al. [42], significant correlations between SPAD values, recorded at anthesis (DC65), with GPC or GLUTEN in mature kernels (DC92) were present (2011: y = 1.75x - 51.5, R² = 0.96 and y = 1.42x - 44.4, R² = 0.96, respectively; 2012: y = 0.58x - 7.9, R² = 0.92 and y = 0.38x - 4.3, R² = 0.87, respectively).

**Fig 6:**
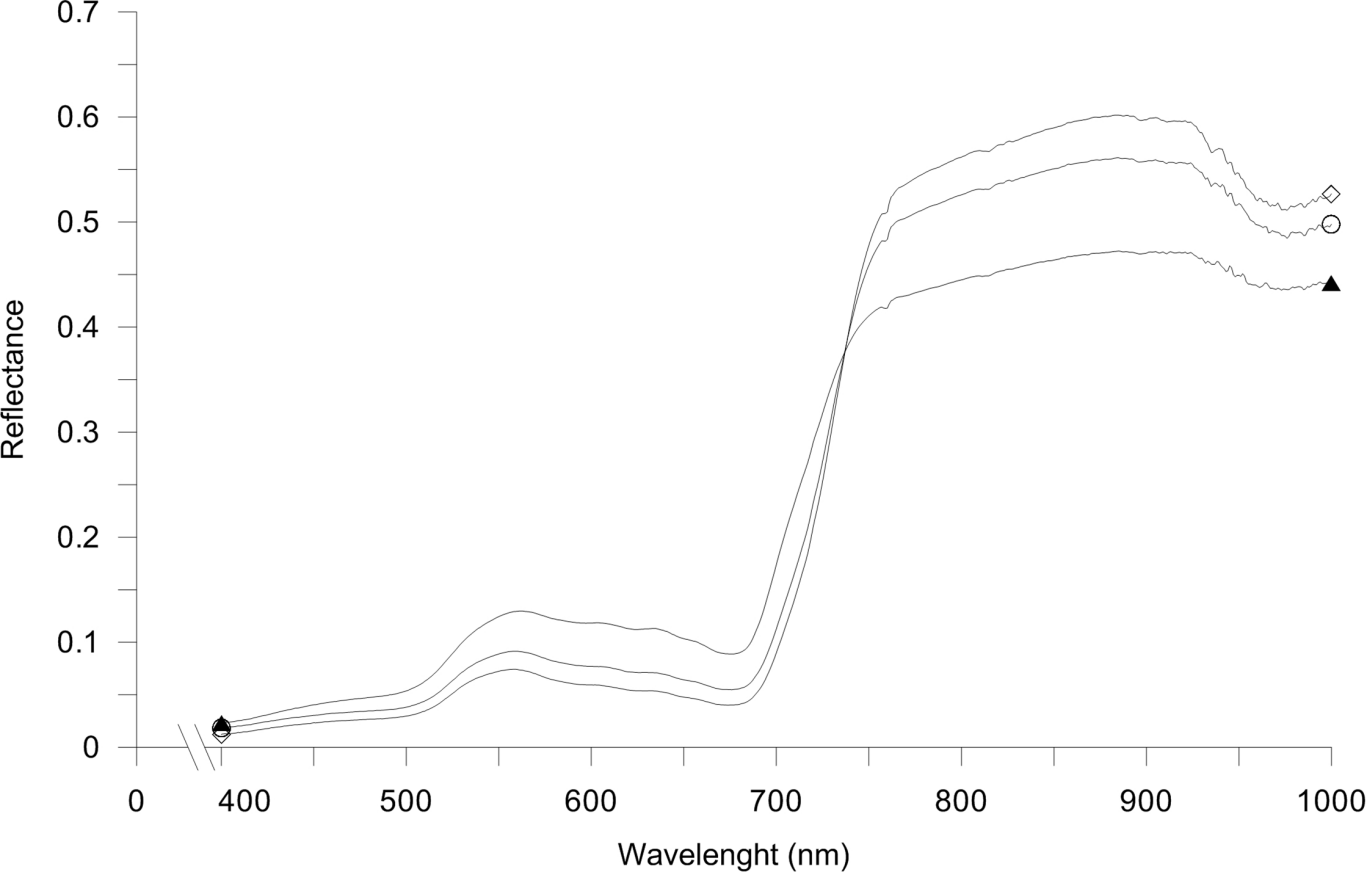
Canopy reflectance of durum wheat plants (averaged over DC65, DC71, DC75 and DC77 phenological stages). Durum wheat plants were exposed to control conditions (unfertilized CONTROL, black triangle) and to N fertilization treatments with urea (UREA, white rhombus) and calcium nitrate (NITRATE, white circle) at the rate of 150 kg N ha^-1^, in 2012.

Vegetation indices (VI*s*) are a useful tool to assess plant N status and, consequently, to estimate GPC at harvest [14]; in this work, a good spectral separability of canopy reflectance was observed among the different treatments, principally in the NIR region (700–1000 nm) (Fig 6), as previously observed [15]. UREA-treated plants showed the highest reflectance values, corresponding with the higher quality trait (i.e. GS-fraction content) at harvest. The correlation coefficients of GPC *vs* VI*s* and GLUTEN *vs* VI*s* at different phenological stages during grain development, are shown in Table 2.

**Table 2:** Pearson’s correlation coefficients between grain protein content (GPC, %) or total gluten protein content (GLUTEN, mg g^-1^ flour) of durum wheat grains at harvest (DC92) and nine VI*s* calculated from reflectance data recorded at different growth stages, during grain-development (DC65, DC71, DC75 and DC77, corresponding to 0, 6, 13 and 21 days post-anthesis (DPA)) in 2012.

	GPC (%)				GLUTEN (mg g^-1^ flour)			
VI*s*	0 DPA	6DPA	13DPA	21DPA	0 DPA	6DPA	13DPA	21DPA
NDVI	.873**	§	.859**	.923**	.906**	§	.887**	.926**
GNDVI	.907**	§	.893**	.941**	.937**	§	.917**	.937**
OSAVI	.882**	§	.891**	.911**	.916**	§	.919**	.922**
SR	.882**	*n*.*s*.	.945**	.925**	.910**	.733*	.950**	.887**
SIPI	- .832**	§	- .812**	- .932**	- .868**	§	- .838**	- .949**
NRI	.816**	.765*	.815**	*n*.*s*.	.843	.789*	.845**	*n*.*s*.
MCARI	- .799**	*n*.*s*.	- .868**	- .844**	- .825**	- .743*	- .893**	- .805**
TVI	.711*	.774*	.894**	.781*	.742*	.764*	.923**	.819**
WI	.883**	.823**	.711*	.921**	.907**	.896**	.807**	.921**

* significant effect at the 0.05 probability level.

** significant effect at the 0.01 probability level. *n*.*s*. = not-significant.

§ = not-applicable correlation test; p-value < 0.05 for Shapiro-Wilk normality test.

Eight reflectance indices were selected to estimate the contents of pigments and other biochemical components in plants (NDVI, GNDVI, OSAVI, SR, SIPI, NRI, MCARI and TVI) [14,15], plus an indicator of plants water status (WI) [43]. In general, all the VI*s* were significantly correlated with both GPC and GLUTEN variables (R^2^ ranges between 0.711 and 0.950) very early during grain filling (DC65, Table 2).

**Fig 7:**
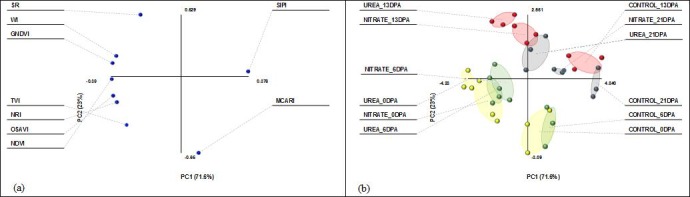
Two dimensional principal component analysis (PCA) in 2012. (a) loading plot for the 9 variables (VIs indices: NDVI, GNDVI, WI, OSAVI, SIPI, NRI, SR, MCARI and TVI); (b) score plot for the 12 treatments (CONTROL_0DPA, CONTROL_6DPA, CONTROL_13DPA, CONTROL_21DPA, UREA_0DPA, UREA_6DPA, UREA_13DPA, UREA_21DPA, NITRATE_0DPA, NITRATE_6DPA, NITRATE_13DPA, NITRATE_21DPA) obtained as a combination of the 3 N treatments (CONTROL, UREA and NITRATE) at 4 sampling dates during grain development (0, 6, 13 and 21 days post-anthesis (DPA)). Further explanations on VI*s*, sampling dates/phenological stages and N fertilization treatments are provided in S2 Table and in the text.

The relationship between the VI*s* and the N fertilization treatments was investigated with the PCA, graphically displayed in Fig 7. The first and second principal components explained 94.6% of the total data variability (71.6 and 23.0% for PC1 and PC2, respectively). Variables were correlated between each other, with the exception of MCARI (0.10 and -0.65 on PC1 and PC2, respectively), TVI (-0.30 and -0.41 on PC1 and PC2, respectively) and NRI (-0.36 and -0.22 on PC1 and PC2, respectively). Interestingly, for each DPA all the VI*s* discriminated significantly between unfertilized and fertilized plots, as well as between UREA and NITRATE (Fig 7B), supporting the observed differences in GS-fractions recorded in 2012.

### LMW-GS protein accumulation in relation to fertilization treatments

**Fig 8:**
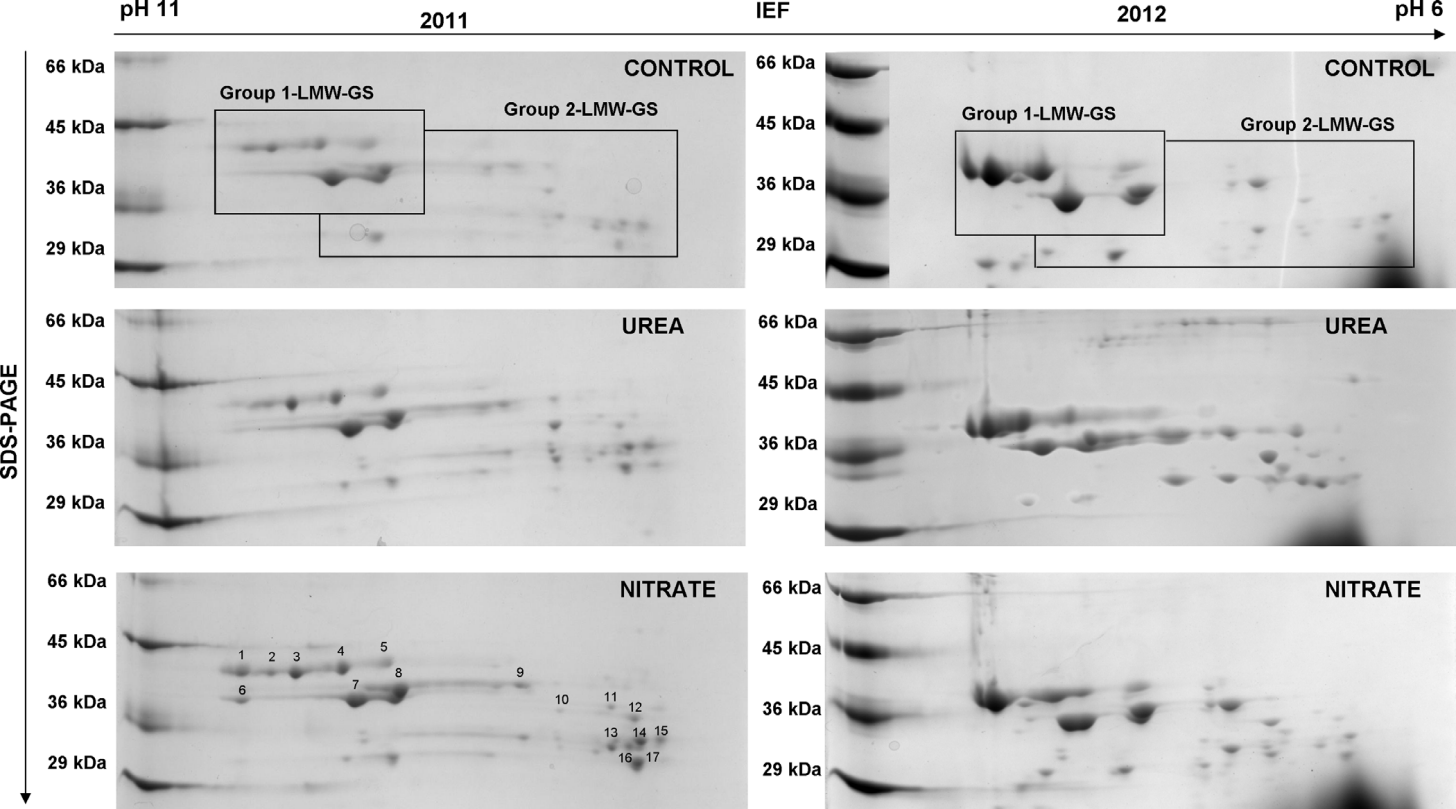
2D-GE pattern of the LMW-GS from durum wheat (cv Achille) in 2011 and 2012. Comparison of 2D-GE maps of the LMW-GS fractions extracted from mature grains of durum wheat fertilized with urea (UREA) and calcium nitrate (NITRATE) at the rate of 150 kg N ha^-1^ plus unfertilized CONTROL in 2011 and 2012. The marked spots were analysed by LC/MS for protein identification.

N fertilization influenced GS-fractions (Fig 4). Consequently, to explain whether the differences in GS contents might be ascribed to specific GS subunits, 2D-GE was performed on LMW-GS samples. Two distinct protein groups, indicated as Group 1 and Group 2 were considered (Fig 8).

Quantitative analysis of selected spots showed the specific induction of Group 2 from N-fertilised samples, regardless of N-form, both in 2011 and 2012 (Table 3).

**Table 3:** Quantitative densitometry analysis of LMW-GS from Group 1 and Group 2 extracted from wheat grains deriving from plants exposed to control conditions (unfertilized CONTROL) and to two fertilization treatments with Urea (UREA) and Calcium Nitrate (NITRATE) at the rate of 150 kg N ha^-1^.

	*2011*		*2012*	
*Treatment*	Group 1 LMW-GS (IOD μg^-1^ protein)	Group 2 LMW-GS (IOD μg^-1^ protein)	Group 1 LMW-GS (IOD μg^-1^ protein)	Group 2 LMW-GS (IOD μg^-1^ protein)
CONTROL	2,299,328.9 b	815,634.8 c	5,807,070.1 b	1,083,857.5 b
UREA	1,812,438.8 c	1,125,028.1 b	8,528,323.1 a	1,774,638.1 a
NITRATE	2,736,053.2 a	1,579,498.6 a	6,446,072.2 b	1,765,693.9 a
*F test*	*	*	*	**

*P < 0.05

**P < 0.01; ns = not-significant. Values with the same letter (a, b, c) are not significantly different (P < 0.01). IOD = integrated optical density.

Sequence analysis of these proteins showed that Group 1 corresponded with LMW-GS present in the basic region of the gel and that Group 2 corresponded with LWM-GS, which are structurally similar to γ-gliadins [44] (Table 4).

**Table 4:** 2D-GE LMW-GS protein identification by LC-ESI-MS/MS analysis.

Spot n.^a^	Protein identified	Species	UniProtK Acc. nr.	MW	Peptide sequences determined by LC-ESI-MS/MS	Measured MH^+^
**Group 1**						
1	LMW-GS GluB3-6	*T*. *aestivum*	B2Y2R3	44613	LQPHQIAQLEVMTSIAL,VHPSILQQLNPCKVF, RTLPTMCNVNVPL	1892.035, 1779.957, 1514.783
2	LMW-GS GluB3-6	*T*. *aestivum*	B2Y2R3	44613	LQPHQIAQLEVMTSIAL, VHPSILQQLNPCKVF,RTLPTMCNVNVPL	1892.035, 1779.957, 1514.783
3	LMW-GS GluB3-3	*T*. *durum*	D5FPE1	44540	VPFGVGTGVGGY, VFLQQQCSPVAMPQSLAR, SQMLQQSSCHVMQQQCCQQLPQIPQQSR	1109.562, 2003,020, 3272.4850
4	LMW-GS B3-2	*T*. *aestivum*	Q6SPY7	44702	MCSVNVPLYETTTSVPLGVG IGVGVY, SQMLQQSICHVMQR, VFLQQQCIPVAMQR	2712.3622, 1745.8244, 1717.8876
5	LMW-GS B3-2	*T*. *aestivum*	D3U319	44509	VHPSILQQLNPcKVF, RTLPTMcNVNVPL, GQQPQQQQLAHGTF, RTLPTmcNVNVPLY, QQQIPFVHPSIL	1476.799, 1301.659^b^, 1567.761 ^b^, 1464.722 ^b^^,^^c^, 1406.779
6	LMW-GS	*T*. *aestivum*	R4JDM5	37783	SIVLQEQQHGQGLNQPQQQQPQQSVQGVSQPQQQQKQLGQcSF QSScHVMQQQccRQLPQIPEQSRY, RTLPTMcSVNVPVYGTTTIVPF, METSHIPSLEKPLQQQPLPL, LQQQcSPVAmPQSL, GQWPQQQQVPQGTLLQPHQIAQLEVMTSIAL	4884.392 ^b^, 3048.374 ^b^, 2453.262 ^b^, 2286.221, 1602.765 ^b^^,^^c^, 3467.828
7	LMW-GS	*T*. *aestivum*	R4JDM5	37783	SIVLQEQQHGQGLNQPQQQQPQQSVQGVSQPQQQQKQLGQcSF, QSScHVMQQQccRQLPQIPEQSRY, RTLPTMcSVNVPVYGTTTIVPF, METSHIPSLEKPLQQQPLPL, LQQQcSPVAmPQSL, GQWPQQQQVPQGTLLQPHQIAQLEVMTSIAL	4884.392 ^b^, 3048.374 ^b^, 2453.262 ^b^, 2286.221, 1602.765 ^b^^,^^c^, 3467.828
8	LMW-GS	*T*. *aestivum*	R4JDM5	37783	SIVLQEQQHGQGLNQPQQQQPQQSVQGVSQPQQQQKQLGQcSF, QSScHVMQQQccRQLPQIPEQSRY;, RTLPTMcSVNVPVYGTTTIVPF, METSHIPSLEKPLQQQPLPL, LQQQcSPVAmPQSL, GQWPQQQQVPQGTLLQPHQIAQLEVMTSIAL	4884.392 ^b^, 3048.374 ^b^, 2453.262 ^b^, 2286.221, 1602.765 ^b^^,^^c^, 3467.828
**Group 2**						
9	γ-gliadin	*T*. *aestivum*	R9XUS6	40767	SLVLQTLPTMCNVYAPPECS TIR, APFASIVAGIGGQ	2536.2608, 1187.6418
10	γ-gliadin	*T*. *aestivum*	K7X1Q2	34372	GIIQPQQPAQLEGIRSL, cEQPQRTIPQPHQTF, LQQQmNPcKNY	1848.038, 1866.895, 1439.643 ^b^^,^^c^
11	γ-gliadin	*T*. *aestivum*	D0EMA4	34280	FQLAQGLGIIQPQQPAQLEGIRSL, LQQQMNPcKNF, cEQPQRTIPQPHQTF, VLKTLPTmcNVY, QcAAIHSVAHSIIMQQEQQQGVPILRPL	2605.457, 1407.653, 1866.895, 1242.588 ^b^^,^^c^, 3152.656
12	γ-gliadin	*T*. *aestivum*	K7X1Q2	34372	GIIQPQQPAQLEGIRSLVL, VLKTLPTmcNVYVPPDcSTINVPY, cEQPQRTIPQPHQTF, LQQQMNPcKNY	2060.191, 2797.369 ^b^^,^^c^, 1423.648, 1242.587
13	γ-gliadin	*T*. *durum*	Q84M19	32195	GIIQPQQPAQLEGIRSL, cEQPQRTIPQPHQTF, VLKTLPTmcNVYVPPDcSTINVPY, ANIDAGIGGQ	1848.040, 1866.896 ^b^, 2797.369 ^b^^,^^c^, 915.455
14	γ-gliadin	*T*. *durum*	Q84M19	32195	GIIQPQQPAQLEGIRSLVL, cEQPQRTIPQPHQTF, LQQQmNPcKNF, KTLPTmcNVYVPPDcSTINVPY	2060.192, 1866.899, 915.455 ^b^^,^^c^, 2569.223 ^b^^,^^c^
15	γ-gliadin	*T*. *durum*	Q84M19	32195	GIIQPQQPAQLEGIRSL, cEQPQRTIPQPHQTF, ANIDAGIGGQ, KTLPTMcNVYVPPDcSTINVPY	1848.040, 1866.899, 915.455, 2569.223
16	LMW-GS	*T*. *aestivum*	R4JFH1	30123	VQAQQQQPQQLGQGVSQSQQQSQQQLGQcSF, SIILQEQQQGF, LQQQcNPVAmPQRL, SQQQLVLPPQQQYQQVLQQQIPIVQPSVL, SQQQQPVLPQQSPF, DAIRAITY	3486.656, 1290.672, 1698.846 ^b^^,^^c^, 3355.846, 1611.818, 922.502
17	LMW-GS	*T*. *aestivum*	R4JFH1	30123	VQAQQQQPQQLGQGVSQSQQQSQQQLGQcSF, SIILQEQQQGF, RTLPTmcSVNVPLYSSTTSVPF,QQSScHVmQQQccQQLPQIPEQSRY, QQLNPcKVF	3486.667 ^b^, 1290.671, 2473.214 ^b^^,^^c^, 3355.846 ^b^^,^^c^, 1133.578 ^b^

^a^ Spots numbers in the different treatments as indicated in 2D-GE.

^b^ Cystein carbamidomethylation.

^c^ Methionin oxidation.

*In silico* alignments of the putative protein sequences of these two groups showed the same number of cystein residues at the C-term region of the proteins, six of them being present in a conserved position in all the GS sequenced (S1 Fig).

Group 1 LMW-GS showed differences between treatments in the two years. In 2011, NITRATE treatment showed more Group 1 LMW-GS than the CONTROL while, in 2012, the highest content of Group 1 LMW-GS was achieved by UREA treatment. Therefore, an increase in LMW-GS in response to N fertilization was principally accounted for by Group 2 LMW-GS while differences in abundance of Group 1 LMW-GS was more related to environmental and seasonal variations. Other recent proteomic data underlined the induction of specific components of gluten proteins (HMW-GS, LMW-GS and gliadins) in response to N fertilization both in the developing endosperm and in wheat flour [6,7]. The combined effect of temperature and fertilization could affect the expression of individual proteins within certain classes, such as LMW-GS and gliadins, thus increasing the data complexity [35].

### N treatments and total protein accumulation during grain development

**Fig 9:**
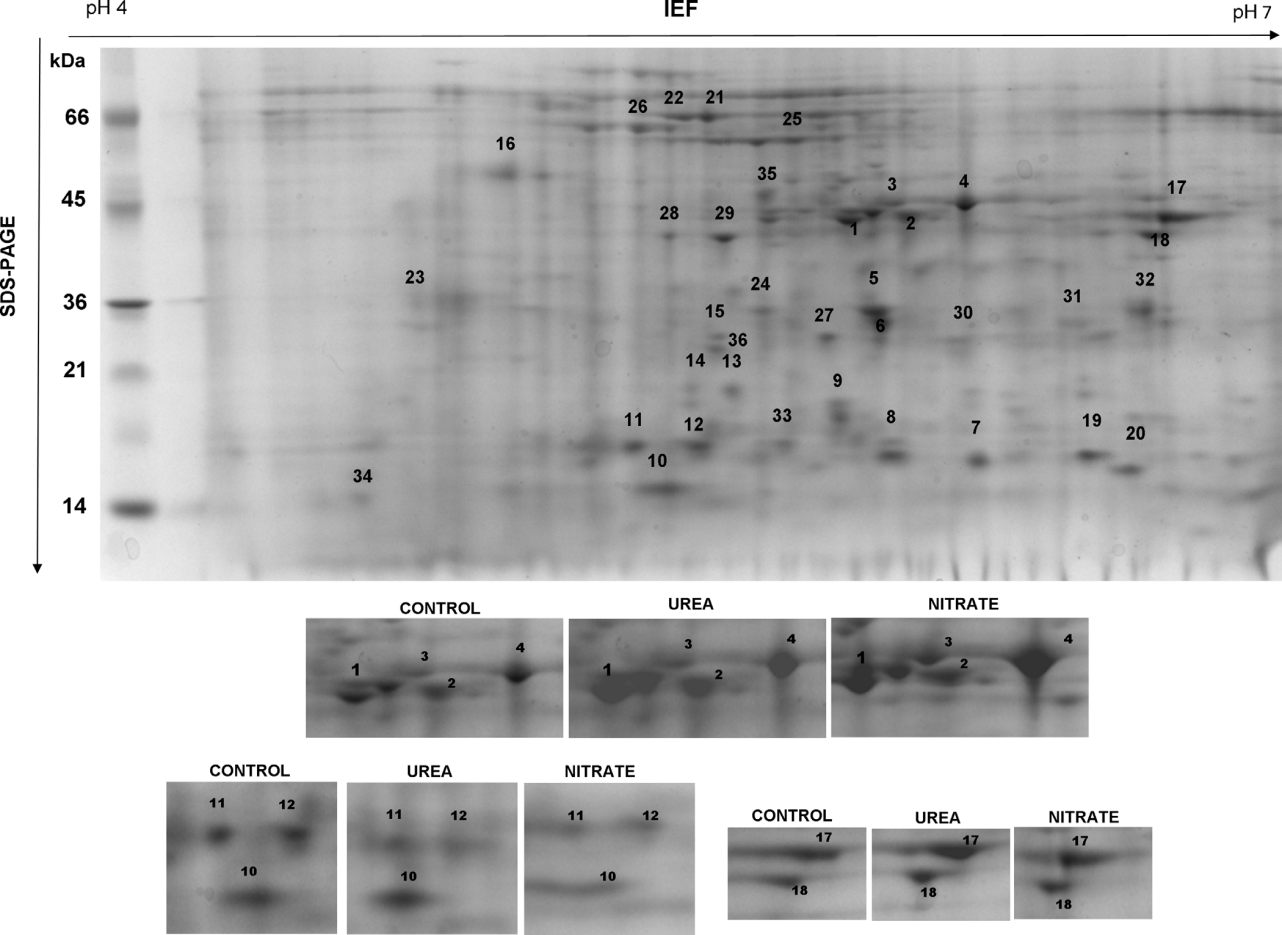
2D-GE pattern of total protein extracted from durum wheat (cv Achille) immature grains. Immature grains—derived from plants fertilized with urea (UREA) and calcium nitrate (NITRATE) at the rate of 150 kg N ha^-1^ plus unfertilized CONTROL—were sampled at DC75 phenological stage corresponding to 15 DPA and 13 DPA in 2011 and 2012 and total protein extracts were analysed by 2D-GE. The marked spots were analysed by ESI-LC-MS/MS for protein identification. The enlargement illustrates some of the spots showing differential abundance between CONTROL and UREA and NITRATE fertilized treatments.

Total proteins extracted from immature grains, collected at 15 DAP in 2011 and at 13 DAP in 2012 (DC75), were separated by 2D-GE (Fig 9), to identify differences due to N fertilization in the amount of grain proteins due to N fertilization during the active phase of grain filling.

**Fig 10:**
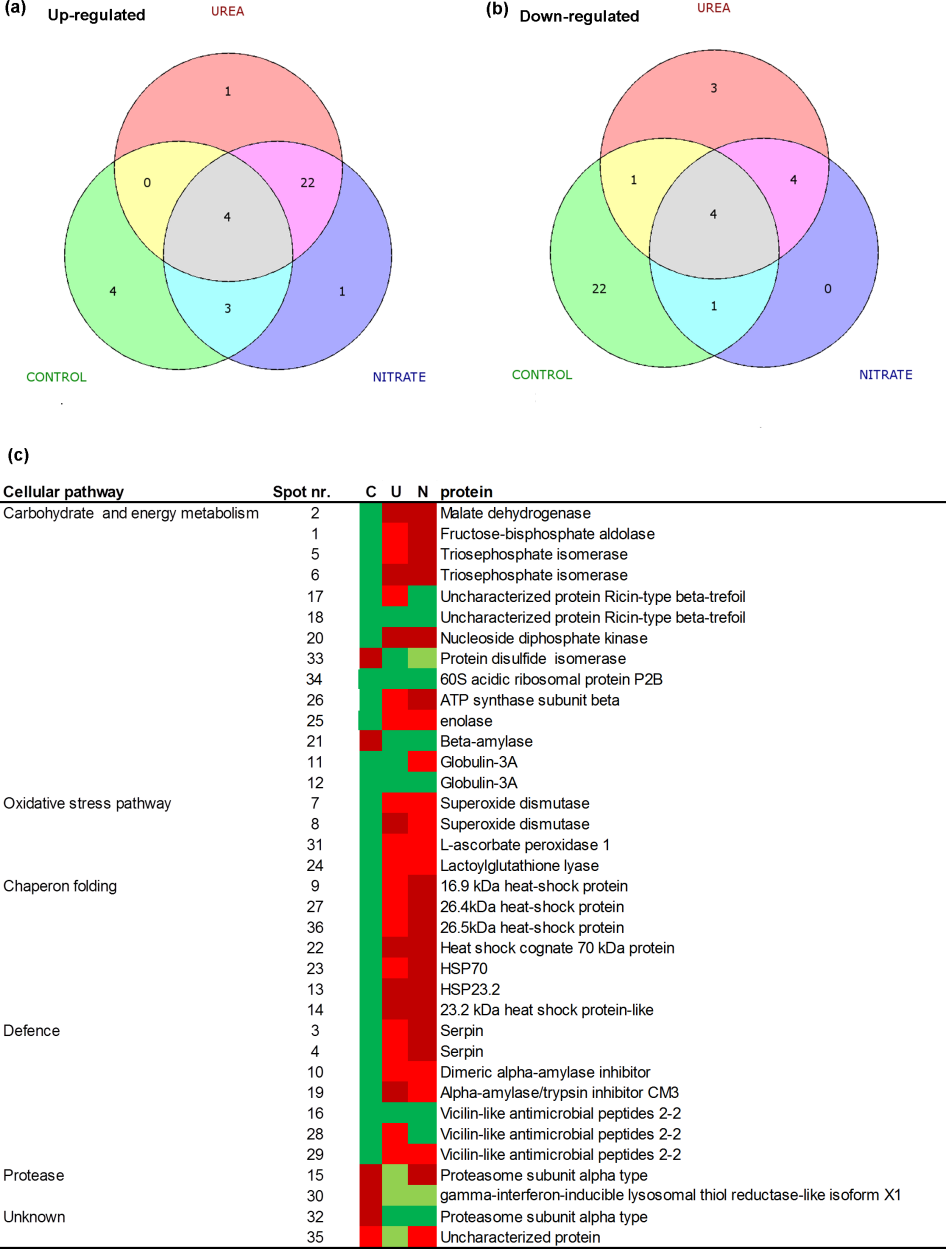
ESI-LC-MS/MS identification and differential abundance of proteins from durum wheat immature grains. (a) Venn diagram showing the number of unique proteins in the 15 and 13 DPA (in 2011 and 2012, respectively) phase of grain filling, which were up-regulated in the different treatments, (b) Venn diagram showing the number of unique proteins in the 15 DPA and 13 DPA (in 2011 and 2012, respectively) phase of grain filling, which were down-regulated in the different treatments, (c) Heat map showing protein abundance by UREA (U) and NITRATE (N) fertilization treatments compared with unfertilized CONTROL (C). Data on single protein sequence and function obtained by LC-ESI-MS/MS analyses are listed in S3 Table. Fold variation between data was normalised as follows: up-regulation 2, 3- fold bright red, > 3 fold dull red, down-regulation 2, 3-fold bright green, >3 fold dull green.

According to [45], at this developmental stage, kernels are mainly characterized by globulin and by proteins with specific functions, which participate in various metabolic activities as carbohydrate accumulation and protein folding during grain filling. Consequently, they could be related to wheat quality traits at harvest [2,46]. Moreover, this crop stage matches with the start of the active storage of starch and water [4]. Thirty-five proteins revealed to be unique from the gel spots identified, four of them showing the same level of abundance in all the treatments, while others showing differences in abundance in UREA and NITRATE showing up-regulation respect to CONTROL (Fig 10A and 10B). The putative functions of identified protein spots in biochemical processes during grain filling are presented in Fig 10C and S3 Table.

### Carbohydrate and energy metabolism

Fructose bisphosphate aldolase, triosephosphate isomerase, enolase and malate dehydrogenase (Fig 9, spots 1, 2, 5, 6, 25) are enzymes involved in glycolysis and in the citric acid cycle which were up-regulated in both UREA and NITRATE treatments (Fig 10C). 15 DAP is the stage at which cell differentiation is occurring and dry matter accumulation begins, both of which require energy as shown by the up regulation of ATP synthase (Fig 9, spot 26) in both treatments. Nucleoside diphosphate kinase (NDPK) (Fig 9, spot 20) is a ubiquitous enzyme whose function is the intracellular distribution of terminal phosphate bond energy among the various nucleotides used for biosynthetic pathways and for regulatory functions [47]. Plant NDPKs are also involved in signal transduction, differentiation and development [48]. Results showed that NDPK was up-regulated during grain development in both N treatments (Fig 10C) and, therefore, might play an important role in signal regulation for grain development in response to N fertilization. A β-amylase was down-regulated (Figs 9 and 10C, Spot 21) in both N treatments. B-amylase is a starch-degrading enzyme that hydrolytically cleaves a-1,4-D-glucosidic bonds to liberate β-maltose from the non-reducing ends of a variety of polyglucans that are synthesised during grain development, and it is one of the major proteins in the starchy endosperm [49]. The level of this protein is normally low in the active phase of grain filling while its down-regulation was recently shown to be cultivar specific accounting for the different size of starch granules in mature grains [45]. In this research the down-regulation of this enzyme in developing grains under N treatments respect to control can be explained as a major input for starch granule formation at the beginning phase of grain filling.

During the active grain-filling period, no increase in globulins in response to UREA treatment was observed, consistent with the findings of a previous study [50]; NITRATE induced a significant accumulation of storage proteins (Figs 9 and 10C, spots 11, 12). These proteins are known to accumulate gradually during seed development and disappear during germination, which is concomitant with the acquisition of both seed and seedling vigor.

### Chaperon folding

The abundance of HSP70 and low molecular weight (LMW) HSPs, namely two members of 23.2 HSPs, two members of 26.4 kDa HSPs and one member of 16.9 kDa, increased due to N fertilization (Figs 9 and 10C, spots 9, 13, 14, 22, 23, 27, 36) as also previously reported [51]. Members of the HSP70 gene family are known components of the cellular network of molecular chaperones and are essential in normal cell functions [52]. It has been demonstrated that they increase both in the endosperm and in the embryo in response to high temperature during grain filling [4,53]. LMW HSPs are produced in seeds during maturation and under various stress conditions, which can form large multimeric structures and display a wide range of cellular functions, as well as being able to act as molecular chaperones. LMW HSPs were shown to be up-regulated in heat-tolerant cultivars during mid-grain development [53]. A positive correlation between small HSPs amounts in the developing grain with wheat yield related traits was also assessed recently [45]. Thus cultivar specific enhancement of HSP production, determined also by N fertilization, could be a parameter used to screen for suitable genotypes in the transition to CA. Modulating N inputs, both storage protein contents and heat-tolerance can be increased.

### Antioxidant enzymes

Several proteins were identified with potential roles in response to oxidative stress. The amount of these proteins increased moderately in N-fertilized samples, reflecting their relevance in protecting the seed against a desiccation-induced stress due to the active oxygen species which are produced during seed development. These identified enzymes include ascorbate peroxidase, superoxide dismutase, catalase, and glutathione lyase (Figs 9 and 10C, spots 7, 8, 31, 24,). Several authors have demonstrated that exposure to heat stress during the reproductive phase [54] increases the activities of antioxidant enzymes particularly in heat-tolerant genotypes of wheat [55].

### Protease inhibitors

The induction of anti-oxidative enzymes is normally accompanied by a down-regulation of proteasomes, which we identified as spots 15 and 32 (Figs 9 and 10C). Proteasome is a multicatalytic proteinase complex involved in ATP/ubiquitin-dependent proteolytic pathways which can degrade unneeded or damaged proteins to protect the seed [56]. In addition a γ-interferon responsive lysosomal thiol reductase known to be involved in unfolded protein degradation was also down-regulated (Figs 9 and 10C, spot 30) as indication of the reduced oxidative damage due to environmental constrains in the developing grains under N treatments.

### Defense proteins

Accumulation of starch and of storage proteins was accompanied by the expression of various α-amylase inhibitors (Figs 9 and 10C, spots 10, 19), which are mainly located in plastids or in the extracellular spaces. Alpha-amylase inhibitors play important roles in protecting starch and protein reserves in the endosperm against degradation caused by biotic stress [57]. The identified α-amylase inhibitors were up-regulated in both fertilization treatments, consistent with the accumulation patterns of starch and storage proteins during late grain development. Another group of proteins whose abundance increased during both N fertilization treatments were identified as serpins (Figs 9 and 10C, spots 3, 4), which are thought to have a role as storage proteins in plants due to their high Lys content. Although their biochemical role is still unclear, because no target proteases have been identified, serpins may provide protection against insect and pathogens [58]. Also vicilin-like antimicrobial proteins were moderately induced in particular by UREA (Figs 9 and 10C, spots 28, 29). These storage proteins belong to the cupin superfamily of proteins which have multiple functions [59], providing protection against fungi and bacteria, as found in *Macadamia integrifolia* kernels [60].

## Conclusions

Transition phase to CA represents an holistic change in the management of the agronomic techniques; during this adaptive phase yields of durum wheat could be unstable and reduced compared to conventional-tillage based systems. It is strongly influenced by soil-climatic characteristics, residue management and N fertilization practices where in particular modulation of N inputs needs to be adequately adjusted, combining rate with N-forms. N application induced significant dry matter accumulation in vegetative organs and grains during their development as well as grain protein and total gluten (i.e. HMW-GS and LMW-GS) increasing at harvesting. This occurred thanks to an improved crop physiological status, i.e. higher chlorophyll content, which could be effectively monitored by SPAD and VI*s* methods. Besides, the VI*s* recorded during the earlier stages of grain-filling, demonstrated to be effective indicators of kernels quality traits. The VI*s* significantly discriminated between unfertilized and fertilized treatments, as well as between the two N-forms (see PCA). Specifically, UREA seemed to induce higher LMW-GS accumulation than NITRATE, supported by the lower grain-NutE values, although this occurred only in the second year. In addition, N availability greatly affected the GS fractions rather than gliadins.

Quantitative analysis of LMW-GS fractions, followed by single protein identification showed a stable increase from year to year in C-type LMW-GS structurally similar to γ-gliadins but containing extra cystein residues which enabled them to form intermolecular crosslinks with the other glutenin components, thus acting as LMW-GS from a technological point of view.

Both UREA and NITRATE could also influence, at different extents the non-prolamin component of wheat grains, during the grain filling period increasing their abundance along with metabolic enzymes for energy production and with members of the HMW and LMW HSPs and enzymes involved in antioxidant response and biotic stress defense. These proteins protect against heat stress during grain filling, increasing tolerance and helping protein and starch to accumulate in the mature grains.

The specific up-regulation of some proteins in the early stages of grain development as well as physiological indicators related to fitness traits, could be usefully employed in wheat breeding programs for screening genotypes suitable for the particular situation characterizing the transition phase to CA under Mediterranean environments (dry conditions) i.e. increase in crops weed, pest, and disease pressures, unexpected changes in soil carbon content and a low crop residues production. Such genotypes in combination with appropriate agronomic techniques would improve nitrogen use efficiency and thus ensure high performance in terms of biomass, nitrogen accumulation as well as quality traits of the developing grains.

## Supporting Information

S1 Fig Multiple alignment of the ten LMW-GS protein sequences of Group 1 and Group 2 identified by LC-ESI-MS/MS analysis obtained from UniprotKB. (DOCX)

S1 Table Total rainfall and average maximum and minimum temperatures registered during the growing seasons 2011 and 2012. (DOCX)

S2 Table Details of durum wheat grain’s sampling during the post-anthesis period. (DOCX)

S3 Table Total protein identification by LC-ESI-MS/MS analysis and peptide sequences. (DOCX)

## References

[1] Laino P, Shelton D, Finnie C, De Leonardis AM, Mastrangelo AM, Svensson B, et al (2010) Comparative proteome analysis of metabolic proteins from seeds of durum wheat (cv. Svevo) subjected to heat stress. Proteomics: 2359–2368 10.1002/pmic.200900803 20394079

[2] Osipova SV, Permyakova MD, Permyakov AV (2012) Role of non-prolamin proteins and low molecular weight redox agents in protein folding and polymerization in wheat grains and influence on baking quality parameters. J Agric Food Chem: 60:12065–12073. 10.1021/jf303513m 23170897

[3] Tea I, Genter T, Naulet N, Boyer V, Lummerzheim M, Kleiber D (2004) Effect of foliar sulfur and nitrogen fertilization on wheat storage protein composition and dough mixing properties. Cereal Chem 81:759–766 10.1094/CCHEM.2004.81.6.759

[4] Dupont FM, Altenbach SB (2003) Molecular and biochemical impacts of environmental factors on wheat grain development and protein synthesis. J Cereal Sci 38:133–146 10.1016/S0733-5210(03)00030-4

[5] Kindred DR, Verhoeven TMO, Weightman RM, Swanston JS, Agu RC, Brosnan JM, et al (2008) Effects of variety and fertiliser nitrogen on alcohol yield, grain yield, starch and protein content, and protein composition of winter wheat. J Cereal Sci 48: 46–57 10.1016/j.jcs.2007.07.010

[6] Wan Y, Shewry RP, Hawkesford J (2013) A novel family of γ-gliadin genes are highly regulated by nitrogen supply in developing wheat grain. J Exp Bot 64:161–168 10.1093/jxb/ers318 23162123 PMC3528027

[7] Altenbach SB, Tanaka CK, Hurkman WJ, Whitehand LC, Vensel WH, Dupont FM (2011) Differential effects of a post-anthesis feritiliser regimen on the wheat flour proteome determined by quantitative 2-DE. Proteome Sci 9:46 10.1186/1477-5956-9-46 21816081 PMC3168407

[8] Pittelkow CM, Liang X, Linquist BA, van Groenigen KJ, Lee J, Lundy ME, et al (2015) Productivity limits and potentials of the principles of conservation agriculture. Nature 517: 365–368. 10.1038/nature13809 25337882

[9] Farooq M, Flower KC, Jabran K, Wahid A, Siddique KHM (2011) Crop yield and weed management in conservation agriculture. Field Crops Res 117: 172–183. 10.1016/j.still.2011.10.001

[10] Derpsch R (2008) No-tillage and Conservation Agriculture: A Progress Report. In: Goddard T, Zoebisch M, Gan Y, Ellis W, Watson A, Sombatpanit S (eds) No-Till Farming Systems.World Association of Soil and Water Conservation,Special Publication No. 3., WASWAC, Bangkok, pp. 7–39.

[11] Grahmann K, Verhulst N, Peña RJ, Buerkert A, Vargas-Rojas L, Govaerts B (2014) Durum wheat (*Triticum durum* L.) quality and yield as affected by tillage–straw management and nitrogen fertilization practice under furrow-irrigated conditions. Field Crop Res 164: 166–177 10.1016/j.fcr.2014.05.002

[12] Gruber S, Mohring J, Claupein W (2011). On the way towards conservation tillage-soil moisture and mineral nitrogen in a long-term field experiment in Germany. Soil Till Res 115–116:80–87 10.1016/j.still.2011.07.001

[13] Melero S, Lopez-Belido RJ, Lopez-Bellido L, Mufioz-Romero V, Moreno F, Murillo JM (2011) Long-term effect of tillage, rotation and nitrogen fertiliser on soil quality in a Mediterranean vertisol. Soil Till Res 114: 97–107.

[14] Wang ZJ, Wang JH, Liu LY, Huang WJ, Zhao CJ, Wang CZ (2004) Prediction of grain protein content in winter wheat (*Triticum aestivum* L.) using plant pigment ratio (PPR). Field Crop Res 90: 311–321 10.1016/j.fcr.2004.04.004

[15] Apan A, Kelly R, Phinn S, Strong W, Lester D, Butler D, et al (2006) Predicting grain protein content in wheat using hyperspectral sensing of in-season crop canopies and partial least squares regression. Int J Geoinformatics 2: 93–108.

[16] Galieni A, Stagnari F, Visioli G, Marmiroli N, Speca S, Angelozzi G, et al (2016) Nitrogen fertilization of durum wheat: a case of study in Mediterranean area during transition to Conservation Agriculture. Italian J Agron 11: 662 10.4081/ija.2016.662

[17] De Sanctis G, Roggero PP, Seddaiu G, Orsini R, Porter CH, Jones JW (2012) Long-term no tillage increased soil organic carbon content of rain-fed cereal systems in a Mediterranean area. Eur J Agron 40:18–27. 10.1016/j.eja.2012.02.002

[18] Jensen ES, Peoples MB, Boddey RM, Gresshoff PM, Hauggaard-Nielsen H, Alves BJ, et al (2012) Legumes for mitigation of climate change and the provision of feedstock for biofuels and biorefineries. A review. Agron Sustain Dev, 32: 329–364. 10.1007/s13593-011-0056-7

[19] Velthoff GL, Kuikman PJ, Oenema O (2003) Nitrous oxide emission from animal manures applied to soil under controlled conditions. Biol Fertil Soils 37: 221– 230. 10.1007/s00374-003-0589-2

[20] Tenuta M, Beauchamp EG (2003) Nitrous oxide production from granular nitrogen fertilizers applied to a silt loam soil. Can J Soil Sci 83: 521–532.

[21] Zadoks JC, Chang TT, Konzak CF (1974) Decimal code for growth stages of cereals. Weed Res 14:415–421

[22] Barraclough PB, Howarth JR, Jones J, Lopez-Bellido R, Parmar S, Shepherd CE, Hawkesford MJ (2010) Nitrogen efficiency of wheat: genotypic and environmental variation and prospects for improvement. Eur J Agron, 33: 1–11. 10.1016/j.eja.2010.01.005

[23] Rouse JW, Haas RH Jr, Schell JA, Deering DW (1974) Monitoring vegetation systems in the Great Plains with ERTS. Proceedings of ERTS-1 Symp., 3rd, Greenbelt, MD. 10-15Dec. 1973. Washington, DC: NASA SP 351. Vol. 1. pp. 309–317.

[24] Gitelson AA, Merzlyak MN (1996) Signature analysis of leaf reflectance spectra: algorithm development for remote sensing of chlorophyll. J Plant Physiol 148: 495–500.

[25] Rondeaux G, Steven M, Baret F (1996) Optimization of soil adjusted vegetation indices. Rem Sens Environ 55: 95–107.

[26] Pimstein A, Karnieli A, Bansal SK, Bonfil DJ (2011) Exploring remotely sensed technologies for monitoring wheat potassium and phosphorus using field spectroscopy. Field Crop Res 121: 125–135.

[27] Peñuelas J, Filella I, Gamon JA (1995) Assessment of photosynthetic radiation-use efficiency with spectral reflectance. New Phytol 131: 291–296. 10.1111/j.1469-8137.1995.tb03064.x

[28] Schleicher TD, Bausch WC, Delgado JA, Ayers PD (2001) Evaluation and refinement of the nitrogen reflectance index (NRI) for site-specific fertilizer management. In: 2001 ASAE Annual International Meeting, St. Joseph, MI, USA, ASAE Paper No. 01–11151.

[29] Daughtry CST, Walthall CL, Kim MS, De Colstoun EB, McMurtrey JE (2000) Estimating corn leaf chlorophyll concentration from leaf and canopy reflectance. Remote Sens Environ 74: 229–239.

[30] Broge NH, Leblanc E (2001) Comparing prediction power and stability of broadband and hyperspectral vegetation indices for estimation of green leaf area index and canopy chlorophyll density. Remote Sens Environ 76:156–172.

[31] Peñuelas J, Piñol J, Ogaya R, Filella I (1997) Estimation of plant water content by the reflectance water index WI (R900/R970). Int J Remote Sens 18: 2869–2875.

[32] Singh KN, Shepherd WK, Cornish BG (1991) A simplified SDS-PAGE procedure for separating LMW subunits of glutenin. J Cereal Sci 14: 203–208.

[33] Visioli G, Comastri A, Imperiale D, Paredi G, Faccini A, Marmiroli N (2016) Gel-based and gel-free analytical methods for the analysis of HMW-GS and LMW-GS in wheat flour. Food Anal Meth 9: 469–474 10.1007/s12161-015-0218-3

[34] R Development Core Team (2013) R: A Language and Environmental for Statistical Computing. R Foundation for Statistical Computing, Vienna, Austria URL: http://www.R-project.org/.

[35] Hurkman WJ, Tanaka CK, Vensel WH, Thilmony R, Altenbach SB (2013) Comparative proteomic analysis of the effect of temperature and fertiliser on gliadin and glutenin accumulation in the developing endosperm and flour from *Triticum aestivum* L. cv. Butte 86. Proteome Sci 11:8 10.1186/1477-5956-11-8 23432757 PMC3599944

[36] Zhao CX, He MR, Wang ZL, Wang YF, Lin Q (2009) Effects of different water availability at post-anthesis stage on grain nutrition and quality in strong-gluten winter wheat. Comptes Rendus Biologies 332: 759–764. 10.1016/j.crvi.2009.03.003 19632660

[37] Saint Pierre C, Peterson CJ, Ross AS, Ohm JB, Verhoeven MC, Larson M, et al (2008) Winter wheat genotypes under different levels of nitrogen and water stress: Changes in grain protein composition. J Cereal Sci 47:407–416 10.1016/j.jcs.2007.05.007

[38] Bencze S, Veisz O, Bedő Z (2004) Effects of high atmospheric CO_2_ and heat stress on phytomass, yield and grain quality of winter wheat. Cereal Res Commun 32: 75–82.

[39] Garrido-Lestache E, López-Bellido RJ, López-Bellido L (2004) Effect of N rate, timing and splitting and N type on bread-making quality in hard red spring wheat under rainfed Mediterranean conditions. Field Crop Res 85: 213–236 10.1016/S0378-4290(03)00167-9

[40] Stagnari F, Onofri A, Codianni P, Pisante M (2013) Durum wheat varieties in N‐deficient environments and organic farming: a comparison of yield, quality and stability performances. Plant Breeding 132: 266–275 10.1111/pbr.12044

[41] Lemaire G, Gastal F (2009) Quantifying crop responses to nitrogen deficiency and avenues to improve nitrogen use efficiency In: Sadras V.O., Calderini D.F. (Eds.), Crop Physiology: Applications for Genetic Improvement and Agronomy. Academic Press, pp. 171–211.

[42] Matsunaka T, Watanabe Y, Miyawaki T, Ichikawa N (1997) Prediction of grain protein content in winter wheat through leaf color measurements using a chlorophyll meter. Soil Sci Plant Nutr 43: 127–134.

[43] Zhang JH, Xu Y, Yao FM, Wang PJ, Guo WJ, Li L, et al (2010) Advances in estimation methods of vegetation water content based on optical remote sensing techniques. Sci China Technol Sci 53: 1159–1167 10.1007/s11431-010-0131-3

[44] Muccilli V, Cunsolo V, Saletti R, Foti S, Margiotta B, Scossa F, et al (2010) Characterization of a specific class of typical low molecular weight glutenin subunits of durum wheat by a proteomic approach. J Cereal Sci 51: 134–139 10.1016/j.jcs.2009.11.003

[45] Zhang N, Chen F, Huo W, Cui D (2015) Proteomic analysis of middle and late stages of bread wheat (*Triticum aestivum* L.) grain development. Front Plant Sci 6:735 10.3389/fpls.2015.00735 26442048 PMC4569854

[46] Jiang SS, Liang XN, Li X, Wang SL, Lv DW, Ma CY, et al (2012) Wheat drought-responsive grain proteome analysis by linear and nonlinear 2-DE and MALDI-TOF mass spectrometry. Int J Mol Sci 13: 16065–16083 10.3390/ijms131216065 23443111 PMC3546679

[47] Kihara A, Saburi W, Wakuta S, Kim MH, Hamada S, Ito H, et al (2011) Physiological and biochemical characterization of three nucleoside diphosphate kinase isozymes from rice (*Oryza sativa* L.). Biosci Biotechnol Biochem 75:1740–1745 10.1271/bbb.110285 21897044

[48] Tang L, Kim MD, Yang KS, Kwon SY, Kim SH, Kim JS, et al (2008) Enhanced tolerance of transgenic potato plants overexpressing nucleoside diphosphate kinase 2 against multiple environmental stresses. Transgenic Res 17:705–715 10.1007/s11248-007-9155-2 18027101

[49] Vinje M, Willis DK, Duke SH, Henson C (2011) Differential expression of two beta-amylase genes (Bmy1 and Bmy2) indeveloping and mature barley grain. Planta 233: 1001–1010 10.1007/s00425-011-1348-5 21279650

[50] Johansson E, Prieto-Linde ML, Jonsson JO (2001) Effects of wheat cultivar and nitrogen application on storage protein composition and breadmaking quality. Cereal Chem 78: 19–25 10.1094/CCHEM.2001.78.1.19

[51] Wang K, Zhang X, Goatley M, Ervin E (2014) Heat shock proteins in relation to heat stress tolerance of creeping bentgrass at different N levels. PLOSone 9(7): e102914 10.1371/journal.pone.0102914 PMC4106837 25050702

[52] Frydman J (2001) Folding of newly translated proteins in vivo: the role of molecular chaperones. Annu Rev Biochem 70: 603–647 10.1146/annurev.biochem.70.1.603 11395418

[53] Skylas DJ, Cordwell SJ, Hains PG, Larsen MR, Basseal DJ, Walsh BJ, et al (2002) Heat shock of wheat during grain filling: proteins associated with heat-tolerance. J Cereal Sci 35: 175–188.

[54] Zhao H, Dai T, Jiang D, Cao W (2008) Effects of high temperature on key enzymes involved in starch and protein formation in grains of two wheat cultivars. J Agron Crop Sci 194: 47–54 10.1111/j.1439-037X.2007.00283.x

[55] Almeselmani M, Deshmukh P, Sairam R (2009) High temperature stress tolerance in wheat genotypes: role of antioxidant defence enzymes. Acta Agron Hun 57: 1–14 10.1556/AAgr.57.2009.1.1

[56] Sassa H, Oguchi S, Inoue T, Hirano H (2000) Primary structural features of the 20S proteasome subunits of rice (*Oryza sativa*). Gene 250: 61–66. 10854779 10.1016/s0378-1119(00)00190-6

[57] Vensel WH, Tanaka CK, Cai N, Wong JH, Buchanan BB, Hurkman WJ (2005) Developmental changes in the metabolic protein profiles of wheat endosperm. Proteomics 5:1594–1611 10.1002/pmic.200401034 15800972

[58] Østergaard H, Rasmussen SK, Roberts TH, Hjgaard J (2000) Inhibitory serpins from wheat grain with reactive centres resembling glutamine-rich repeats of prolamin storage proteins. Cloning and characterisation of five major molecular forms. J Biol Chem 275: 33272–22279 10.1074/jbc.M004633200 10874043

[59] Gallardo K, Le Signor C, Vandekerckhove J, Thompson RD, Burstin J (2003) Proteomics of *Medicago truncatula* seed development establishes the time frame of diverse metabolic processes related to reserve accumulation. Plant Physiol 133:664–682 10.1104/pp.103.025254 12972662 PMC219042

[60] Marcus JP, Green JL, Goulter KC, Manners JM (1999) A family of antimicrobial peptides is produced by processing of a 7S globulin protein in *Macadamia integrifolia* kernels. Plant J 6:699–719 10.1046/j.1365-313x.1999.00569.x 10571855

