# Supplementary material for: Proteomics of Durum Wheat Grain during Transition to Conservation Agriculture
Source: PLoS One. 2016 Jun 9;11(6):e0156007. doi: 10.1371/journal.pone.0156007 (PMC4900532; doi:10.1371/journal.pone.0156007)

**Fig. SI** Multiple alignment of the ten LMW-GS protein sequences of Group 1 and Group 2 identified by LC-ESI-MS/MS analysis obtained from UniprotKB- identical residues are coloured in a scale of blue according to % of residues in each column that agree with the consensus sequence. Cys residues are evidenced in black boxes
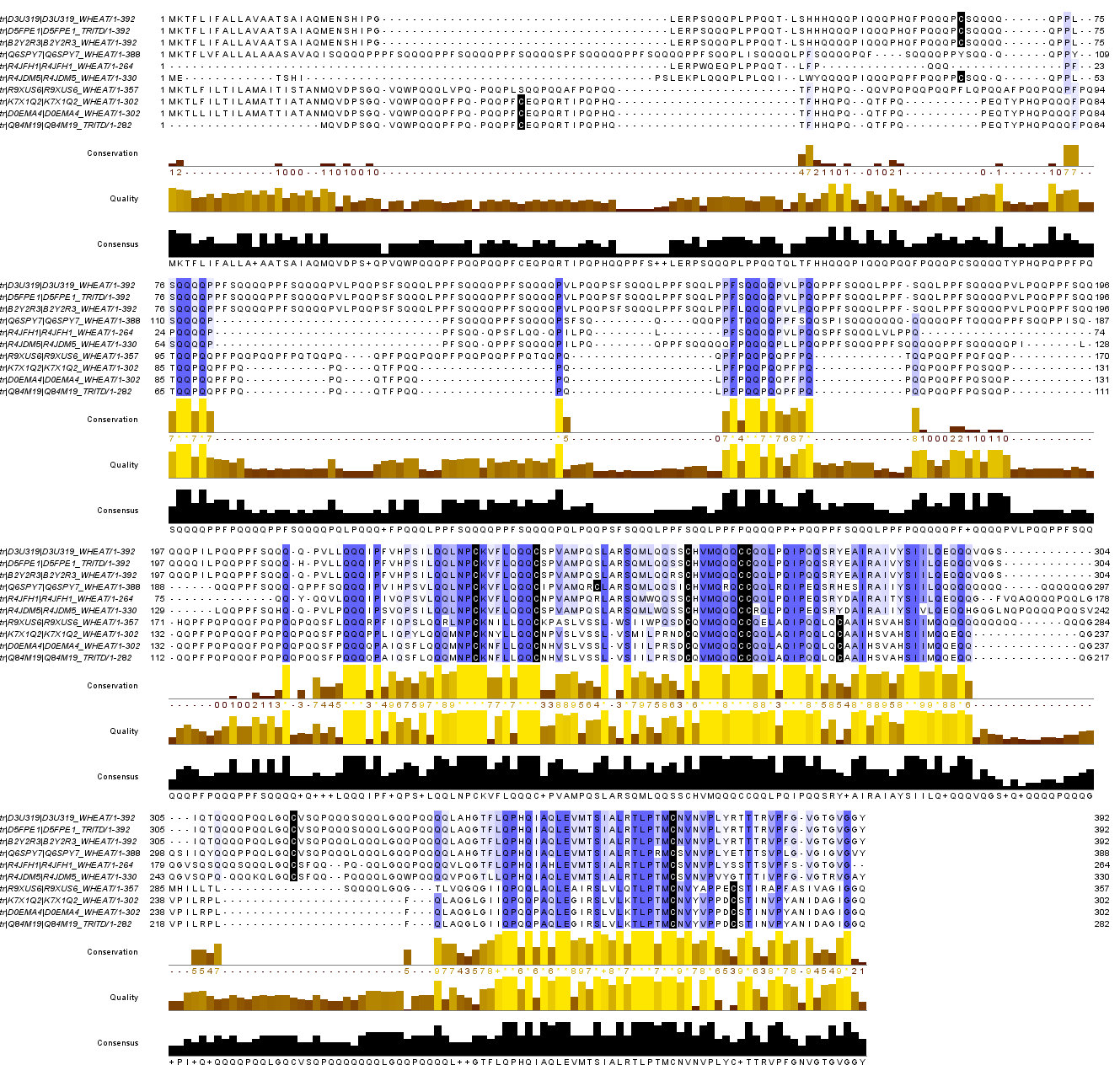

Supplement: S1 Fig — (DOCX) [file pone.0156007.s001.docx]
