# Supplementary material for: Proteomics of Durum Wheat Grain during Transition to Conservation Agriculture
Source: PLoS One. 2016 Jun 9;11(6):e0156007. doi: 10.1371/journal.pone.0156007 (PMC4900532; doi:10.1371/journal.pone.0156007)
Supplement: S1 Table — (DOCX) [file pone.0156007.s002.docx]

Table S1: Total rainfall (mm) and average maximum and minimum temperatures (°C) registered during the growing seasons of 2010/2011 (2011) and 2011/2012 (2012) at Mosciano S. Angelo (TE).

| Month | 2011 | | | | 2012 | | | |
| --- | --- | --- | --- | --- | --- | --- | --- | --- |
|  | T max | T min | Rainfall |  | T max | T min | Rainfall |  |
|  |  |  |  |  |  |  |  |  |
| November | 15.9 | 8.6 | 86.4 |  | 15.2 | 8.1 | 23.8 |  |
| December | 11.2 | 4.1 | 72.2 |  | 13.9 | 6 | 23 |  |
| January | 8.8 | 3.6 | 123.8 |  | 11.1 | 3.2 | 17.6 |  |
| February | 11.2 | 4.5 | 35.4 |  | 7.6 | 1.5 | 141.6 |  |
| March | 12.9 | 6.5 | 182.2 |  | 16.4 | 8.4 | 23.6 |  |
| April | 19.2 | 11.2 | 40.6 |  | 17.8 | 10 | 92.4 |  |
| May | 22.4 | 14 | 32.4 |  | 21.9 | 13.2 | 42 |  |
| June | 26.2 | 18.5 | 38.2 |  | 28.1 | 19.4 | 6.4 |  |
| *Average/Total* | 15.9 | 8.9 | 611.2 |  | 16.5 | 8.7 | 370.4 |  |
