# Supplementary material for: Proteomics of Durum Wheat Grain during Transition to Conservation Agriculture
Source: PLoS One. 2016 Jun 9;11(6):e0156007. doi: 10.1371/journal.pone.0156007 (PMC4900532; doi:10.1371/journal.pone.0156007)
Supplement: S2 Table — (DOCX) [file pone.0156007.s003.docx]

Table S2: Details of durum wheat grain’s sampling during the post-anthesis period. For each sampling date were reported: the phenological development stage scored following the Zadoks Decimal Code (DC), the time expressed in terms of days post-anthesis (DPA) and the thermal time, corresponding to cumulative average daily air temperature exceeding 0 °C, starting from anthesis (DC65).

| Sampling N° | 2011 | | | |  | 2012 | | | |
| --- | --- | --- | --- | --- | --- | --- | --- | --- | --- |
|  | DC | DPA | Δ °Cd^*^ | °Cd |  | DC | DPA | Δ °Cd^*^ | °Cd |
|  |  |  |  |  |  |  |  |  |  |
| I | DC75 | 15 | 243.58 | 243.58 |  | DC75 | 13 | 219.60 | 219.60 |
| II | DC77 | 23 | 141.44 | 385.02 |  | DC77 | 21 | 129.70 | 349.30 |
| III | DC85 | 31 | 170.85 | 555.87 |  | DC85 | 28 | 134.80 | 484.11 |
| IV | DC87 | 39 | 165.58 | 721.45 |  | DC87 | 34 | 123.03 | 607.13 |
|  | | | | | | | | | |
| *** *Cumulative average daily air temperature between two subsequently development stages, starting from anthesis.* | | | | | | | | | |
