# Supplementary material for: Proteomics of Durum Wheat Grain during Transition to Conservation Agriculture
Source: PLoS One. 2016 Jun 9;11(6):e0156007. doi: 10.1371/journal.pone.0156007 (PMC4900532; doi:10.1371/journal.pone.0156007)
Supplement: S3 Table — (DOCX) [file pone.0156007.s004.docx]

Table S4: 2D-GE non-prolamin proteins identification by LC-ESI-MS/MS analysis and their changes in abundance with Urea (UREA, U) and Calcium Nitrate (NITRATE, N) fertilization treatments, at the rate of 150 kg N ha^-1^ with respect to unfertilized CONTROL (C).

| Spot n.^a^ | Protein identified | Species | UniProtK AccessionNr. | MW | Fold difference^b^  U *vs* C N *vs* C | | | | Peptide sequences determined by LC-ESI-MS/MS | Measured MH+ |
| --- | --- | --- | --- | --- | --- | --- | --- | --- | --- | --- |
| Carbohydrate and energy metabolism |  |  |  |  |  |  |  |  | |  |
| 2 | Malate dehydrogenase | *Triticum aestivum* | A3KLL4 | 35500 |  | 3.5 | 3.3 | LGVQVSDVK  VLVTGAAGQIGYALVPmIAR  VLVVANPANTNALILK  GVVATTDVVEAcTGVNVAVmVGGFPR  MELIDAAFPLLK  GVKmELIDAAFPLLK  KKmDATAQELSEEK | | 944.54003  2016.12925^c^  1649.99173  2621.30124^.cd^  1360.75286  1660.93192^c^  1623.78641^c^ |
| 1 | Fructose-bisphosphate aldolase | *Triticum urartu* | M7ZGS6 | 38759 |  | 2.5 | 3.3 | IGATEPSQLSIDQNAQGLAR  GILAADESTGTIGK  KVAPEVIAEYTVR  GTIELAGTNGETTTQGFDDLGKR  ALNDQHVLLEGTLLKPNmVTPGSDAK | | 2069.06028  1332.69878  1474.82380  2381.15567  2777.44790^c^ |
| 5 | Triosephosphate isomerase | *Triticum aestivum* | W5CMD2 | 26757 |  | 2.5 | 3.5 | IKDWTNVVVAYEPVWAIGTGK  IIYGGSVTGAScK  LRPEIQVAAQNcWVK  TNVSPEVAESTR  ELAGQPDVDGFLVGGASLKPEFIDIINAATVK  VIAcVGETLEQREAGSTmAVVAEQTK  RSLmGESSEFVGEK | | 2346.24662  1312.65569  1811.95530^d^  1289.63054  3284.73813  2793.37149^cd^  1571.73520^c^ |
| 6 | Triosephosphate isomerase | *Triticum aestivum* | W5CMD2 | 26757 |  | 3.1 | 3.7 | IKDWTNVVVAYEPVWAIGTGK  LRPEIQVAAQNcWVK  TNVSPEVAESTR  IIYGGSVTGAScK  VAYALAQGLK | | 2346.24662  1811.95530^d^  1289.63103  1312.65569 ^d^  1033.60283 |
| 17 | Uncharacterized protein Ricin-type beta-trefoil; Carbohydrate-binding domain | *Triticum aestivum* | W5DYH0 | 39256 |  | 2.5 | n.s. | ADDGFSATVR  IKDEEGYPAFALVNK  FSTQIKDEEGNPAFAIVNK  ILPWGEEAYAGGSANAPR  DGTSLVLWK | | 1038.48589  1693.88156  2108.06833  1858.91050  1018.55736 |
| 18 | Uncharacterized protein Ricin-type beta-trefoil; Carbohydrate-binding domain | *Triticum aestivum* | W5DYH0 | 39256 |  | n.s. | n.s. | LVPFNPEYLDESVmWTESGDVGK  ILPWGEEAYAGGSANAPR  IKDEEGYPAFALVNK  FSTQIKDEEGNPAFAIVNK  HSLGQSHPVK | | 2628.22153 ^c^  1858.91167  1693.88138  2108.06870  1089.58088 |
| 20 | Nucleoside diphosphate kinase (Fragment) | *Triticum aestivum* | W4ZQ38 | 26063 |  | 3.5 | 4.8 | KEIALWFPEGIAEWR  IIGATNPLASEPGTIR | | 1844.97104  1609.89324 |
| 33 | Protein disulphide isomerase (Fragment) | *Triticum aestivum* | Q6JAB8 | 11570 |  | 3.6 | 2.7 | YEVQGFPTLK  HDPAIVLAK  EAEGIVEYLK | | 1181.61882  963.56053  1150.59795 |
| 34 | 60S acidic ribosomal protein P2B | *Triticum urartu* | M8A407 | 11497 |  | n.s. | n.s. | LIAAYLLAYLGGNSSPSAADVK  FAAVPSGGGAIAVGAPAAASGGAAAPAAESK  GKDITEVIASGR | | 2194.17386  2552.31058  1245.67865 |
| 25 | enolase | *Triticum aestivum* | W5FPN2 | 48056 |  | 2.7 | 2.6 | GNPTVEVDVccSDGTFAR  AAVPSGASTGVYEALELRDGGSDYLGK  LAmQEFmILPTGAASFK  SGETEDTFIADLAVGLSTGQIK  HAGWGVmTSHR  IEEELGAAAVYAGLK | | 1983.85381^d^  2683.32119  1886.93808^c^  2252.13091  1254.57996^c^  1533.81645 |
| 11 | Globulin-3A | *Triticum aestivum* | I6QQ39 | 66284 |  | n.s. | 3.0 | RGSGSESEEEQDQQRYETVR  AKDQQDEGFVAGPEQQQEHERGDR  GSSNLQVVcFEINAERNER  NNVIAKLDDPAQELAFGRPAR | | 2370.05776  2754.24770  2222.06313  2295.22304 |
| 12 | Globulin-3A | *Triticum aestivum* | I6QQ39 | 66284 |  | n.s. | n.s. | RGSGSESEEEQDQQRYETVR  GSSNLQVVcFEINAERNER | | 2370.05849  2222.06467^d^ |
|  |  |  |  |  |  |  |  |  | |  |
| 21 | Beta-amylase | *Triticum urartu* | M8B5G5 | 58709 |  | 4.5 | 3.7 | SGPELTIEmILQAAQPK  LSNQLVEGQNYVNFK  mHANLPHDPcVDPVAPLQR  EGLNmAcENALPR | | 1841.96904^c^  1752.89018  2183.04897^.cd^  1490.67266^cd^ |
| Oxidative stress pathway |  |  |  |  |  |  |  |  | |  |
| 7 | Superoxide dismutase | *Triticum aestivum* | W5B1E5 | 15111 |  | 3.0 | 2. 5 | GTIFFTQEGEGPTTVTGSVTGLK  AVVVHGDADDLGK  AVAVLTGSEGVK  HAGDLGNVTAGVDGVASINITDcHIPLTGPNSIVGR | | 2327.17453  1295.65788  1130.64031  3597.80337^d^ |
| 8 | Superoxide dismutase | *Triticum aestivum* | W5ECA4 | 16960 |  | 3.4 | 2.5 | RGSGSESEEEQDQQRYETVR  AKDQQDEGFVAGPEQQEQERGDR | | 2370.04905  2617.18300 |
| 31 | L-ascorbate peroxidase 1, cytosolic | *Triticum urartu* | M7ZQM4 | 27447 |  | 2.5 | 2.8 | LAWHSAGTFDVSSK  QmGLSDQDIVALSGGHTLGR  TYPVVSAEYQEAVEK  TGGPFGTmK | | 1505.73685  2071.02390  1712.83721  911.42821 ^c^ |
| 24 | Lactoylglutathione lyase | *Triticum urartu* | W5H7T0 | 32653 |  | 2.5 | 2.5 | SAEAVLEWPKQDK  ITSFLDPDGWK  GPTPEPLcQVmLR | | 1500.76921  1278.63579  1513.75005^cd^ |
| 26 | ATP synthase subunit beta | *Triticum aestivum* | Q41534 | 59211 |  | 2.6 | 3.5 | TVLImELINNVAK  LVLEVAQHLGENVVR  mLSPHVLGVDHYNTAR  VLNTGSPITVPVGR  QISELGIYPAVDPLDSTSR  IPSAVGYQPTLATDLGGLQER | | 1473.83415^c^  1675.94858  1825.90226^c^  1409.81157  2061.05132  2186.14653 |
|  |  |  |  |  |  |  |  |  | |  |
| Chaperon folding |  |  |  |  |  |  |  |  | |  |
| 9 | 16.9 kDa heat-shock protein | *Triticum turgidum subsp. durum* | B2FH42 | 16960 |  | 3 | 3.6 | SIVPAISGGSSETAAFANAR  AGLENGVLTVTVPK  KEEVKVEVEDGNVLVVSGER  FRLPEDGKVEEVK | | 1905.96494  1397.79883  2214.15891  1545.82468 |
| 27 | 26.4kDa heat-shock protein | *Triticum dicoccoides* | A5A8U9 | 26430 |  | 2.5 | 3.1 | HAGFDISPFGLVDPmSPmR  VmVEGDALVIR  QmLDTmDRLFDDAVGFPTAR  TmRQmLDTmDRLFDDAVGFPTAR  RSPAAASETPRmPWDImEDDKEVK  NGVLLVSVPK | | 2105.98130^c^  1217.65715^c^  2331.07889^c^  2735.26088^c^  2791.30752^c^  1025.63677 |
| 36 | 26.5kDa heat-shock protein | *Triticum turgidum subsp. durum* | A5A8V2 | 26502 |  | 2.5 | 3.3 | AGFDISPFGLVDPmSPmR  QmLDTmDRLFDDAVGFPTAR  NGVLLVSVPK  LFDDAVGFPTAR  LALPDEcDKSQVR | | 1968.92424^c^  2331.07834^c^  1025.63628  1308.65947  1530.7609^d^ |
| 22 | Heat shock cognate 70 kDa protein | *Triticum urartu* | M7ZPC7 | 71788 |  | 3.3 | 3.6 | EQVFSTYSDNQPGVLIQVYEGER  mVQEAEKYKSEDEQVR  mYQGAGPGGAAGmDEDmPGGGAGTGGGSGAGPK  MVQEAEKYKSEDEQVR  NALENYAYNMR | | 2658.26860  1984.92899^d^  2871.16349^c^  1968.93271  1358.61565 |
| 23 | HSP70 | *Triticum aestivum* | W5ADZ6 | 36515 |  | 3.0 | 3.5 | EQVFSTYSDNQPGVLIQVYEGER  TKDNNLLGK  LSKEEIEKmVQEAER | | 2658.27024  1002.55780  1834.92148^c^ |
| 13 | HSP23.2 | *Triticum aestivum* | W5AT01 | 23925 |  | 3.5 | 4.7 | LPDNADLDSIAASLDAGVLTVR  VVGIAGGDDSSAAK  KTIGDAGAAGGEER  GSRDDAVASPLQDVALLADPFR  ILEHVPFGFDRDDVAmVSmAR | | 2226.16167  1246.62688  1331.65418  2313.18436  2437.16445^c^ |
| 14 | 23.2 kDa heat shock protein-like | *Brachypodium distachyon* | W5AT01 | 23925 |  | 3.6 | 4.2 | QmRLPDNADLDSIAASLDAGVLTVR  GSRDDAVASPLQDVALLADPFR  LPDNADLDSIAASLDAGVLTVR  VVGIAGGDDSSAAK | | 2657.36039^c^  2313.18747  2226.16568  1246.62834 |
|  |  |  |  |  |  |  |  |  | |  |
|  |  |  |  |  |  |  |  |  | |  |
|  |  |  |  |  |  |  |  |  | |  |
| Defence |  |  |  |  |  |  |  |  | |  |
| 3 | Serpin | *Triticum aestivum* | Q41593 | 43091 |  | 2.5 | 3.5 | VSSVFHQAFVEVNEQGTEAAASTAIK  AAEVTTQVNSWVEK  ISFGIEASDLLK  SAASNAAFSPVSLYSALSLLAAGAGSATR  YKAETQSVDFQTK  IKDILPPGSIDNTTK | | 2720.35024  1531.77361  1587.85636  2711.40104  1544.75737  1611.89334 |
| 4 | Serpin | *Triticum aestivum* | Q9ST58 | 42854 |  | 2.8 | 3.5 | VSSVFHQAFVEVNEQGTEAAASTAIK  ISFETEASDLLK  IKDILPSGSVDNTTK  AAEVATQVNSWVEK  LASTISSNPK | | 2720.35024  1352.69353  1587.85636  1531.77361  1017.55644 |
| 10 | Dimeric alpha-amylase inhibitor (Fragment) | *Triticum dicoccoides* | A4GFP2 | 13182 |  | 2.9 | 2.5 | SGPWmcYPGQAFQVPALPGcRPLLK  LPIVVDASGDGAYVcK  LQcNGSQVPEAVLR  DccQQLADISEWcR  LPIVVDASGDGAYVcKDVAAYPDA  EHGVSEGQAGTGAFPScR | | 2846.39297  1663.83451^d^  1570.79924  1840.74028^d^  2466.18496  1846.81180^d^ |
| 19 | Alpha-amylase/trypsin inhibitor CM3 | *Triticum aestivum* | P17314 | 18209 |  | 3.5 | 2.4 | YFIALPVPSQPVDPR  LPEWmTSASIYSPGKPYLAK  SGNVGESGLIDLPGcPR | | 1698.92375  2255.14267^c^  1727.83879^d^ |
|  |  |  |  |  |  |  |  |  | |  |
|  |  |  |  |  |  |  |  |  | |  |
| 16 | Vicilin-like antimicrobial peptides 2-2 | *Triticum urartu* | W5DXK9 | 66395 |  | n.s. | n.s. | RGGDHGQEGVEcK  VKEGDVFVVPR | | 1428.62876  1244.70188 |
| 28 | Vicilin-like antimicrobial peptides 2-2 | *Triticum urartu* | W5E0G5 | 554351 |  | 2.0 | n.s. | TLFVPQYIDSNLILFVQR  LHIIcSIDASDSAGFAPYQSFYLGGGGK  GLFLLDKVEK  VVESEGGSVHVVR  EGLmHIGFITmEPK | | 2166.19571  2931.39768^d^  1161.68730  1353.71062  1634.79123^c^ |
| 29 | Vicilin-like antimicrobial peptides 2-2 | *Triticum urartu* | W5E0G4 | 554351 |  | 2.0 | 2 | TLFVPQYIDSNLILFVQR  GLFLLDKVEK  VVESEGGSVHVVR  EGLmHIGFITmEPK  QTSVLAGFEPK | | 2166.19571  1161.68730  1353.71062  1634.79123^c^  1176.62493 |
| Protease |  |  |  |  |  |  |  |  | |  |
| 15 | Proteasome subunit alpha type | *Brachypodium distachyon* | I1IBV9 | 25796 |  | 2.8 | n.s. | KLPSILVDETSVQK  LVQIEHALTAVGSGQTSLGIK  LYKETIPVTQLVR  AANGVVIATEK | | 1556.88974  2122.18906  1559.91629  1072.60076 |
| 30 | gamma-interferon-inducible lysosomal thiol reductase-like isoform X1 | *Brachypodium distachyon* | W5DWP8 | 25085 |  | 2.5 | 2.0 | VHLGFIYcVSDLVmK  HREWEScFQK  VHVAIYYESLcPYSAR  GHNLSLEYGR  DGLLDAADLTLVPYGNAK | | 1796.90659^cd^  1406.62552^d^  1927.93686^d^  1145.56877  1845.95853 |
| 32 | Proteasome subunit alpha type | *Triticum aestivum* | W5EMA7 | 27419 |  | 5.6 | 3.5 | VVAEGEAGTVTATDVADAAGTVYR  SPQSIITYNPDQKKEEK  QGFGVSAEVVEAIR  VTYIQEGGSETSSLEVQR | | 2323.14182  1490.74761  1461.76909  3369.63242 |
| Unknown function |  |  |  |  |  |  |  |  | |  |
| 35 | Uncharacterized protein | *Triticum urartu* | T1MVG6 | 41474 |  | 2.4 | n.s. | YVDAVLTIPK  VIKVPEGFDYELYNR  CYISLSEQVKEK  EGAPTAVSHGLWLNIPDYDAPTQmVKPR | | 1118.64385  1841.94083  1483.74387  3079.52993^c^ |

^a^ Spots numbers in the different treatment as indicated in 2D-GE

^b^ Fold variation in spot abundance between the control and the two different fertilization treatments. n.s. indicated that for the corresponding spot was not possible to identify a change that exceeds both a fold variation >2 and a P<0.05

^c^ Methionin oxidation

^d^Cystein carbamidomethylation
